# Brain extraction using the watershed transform from markers

**DOI:** 10.3389/fninf.2013.00032

**Published:** 2013-12-09

**Authors:** Richard Beare, Jian Chen, Christopher L. Adamson, Timothy Silk, Deanne K. Thompson, Joseph Y. M. Yang, Vicki A. Anderson, Marc L. Seal, Amanda G. Wood

**Affiliations:** ^1^Developmental Imaging, Murdoch Childrens Research InstituteMelbourne, VIC, Australia; ^2^Stroke and Aging Research Group, Department of Medicine, Southern Clinical School, Monash UniversityMelbourne, VIC, Australia; ^3^Department of Paediatrics, University of MelbourneVIC, Australia; ^4^Victorian Infant Brain Studies, Murdoch Childrens Research InstituteMelbourne, VIC, Australia; ^5^Florey Department of Neuroscience and Mental Health, University of MelbourneVIC, Australia; ^6^Department of Neurosurgery, Royal Childrens HospitalMelbourne, VIC, Australia; ^7^Clinical Sciences, Murdoch Childrens Research InstituteMelbourne, VIC, Australia; ^8^School of Psychology, University of BirminghamEdgbaston, UK

**Keywords:** brain extraction, scalping, watershed transform from markers, human brain extraction, macaque brain extraction, mathematical morphology, Insight Toolkit

## Abstract

Isolation of the brain from other tissue types in magnetic resonance (MR) images is an important step in many types of neuro-imaging research using both humans and animal subjects. The importance of brain extraction is well appreciated—numerous approaches have been published and the benefits of good extraction methods to subsequent processing are well known. We describe a tool—the *marker based watershed scalper* (MBWSS)—for isolating the brain in T1-weighted MR images built using filtering and segmentation components from the Insight Toolkit (ITK) framework. The key elements of MBWSS—the watershed transform from markers and aggressive filtering with large kernels—are techniques that have rarely been used in neuroimaging segmentation applications. MBWSS is able to reliably isolate the brain without expensive preprocessing steps, such as registration to an atlas, and is therefore useful as the first stage of processing pipelines. It is an informative example of the level of accuracy achievable without using priors in the form of atlases, shape models or libraries of examples. We validate the MBWSS using a publicly available dataset, a paediatric cohort, an adolescent cohort, intra-surgical scans and demonstrate flexibility of the approach by modifying the method to extract macaque brains.

## 1. Introduction

Isolation of the brain from surrounding tissues in magnetic resonance (MR) images of the head, variously referred to as *scalping*, *skull stripping*, *brain extraction* or *brain segmentation*, is a critical step in many forms of analysis. In some cases the scalping step is performed in conjunction with brain tissue classification while in others it is a separate preprocessing step. Precise segementation of the brain from surrounding tissue makes many standard processing steps much simpler and more accurate—widely used software packages such as FreeSurfer (Dale and Fischl, 1999) and FSL (Jenkinson et al., 2012) use explicit skull stripping steps early in their procedures. An isolated brain is a much more useful target for standard processing steps, such as registration, inhomogeneity correction and tissue classification, than the raw MR image. The wide variation in anatomy and tissue contrasts make scalping a surprisingly difficult task to automate reliably and accurately. The difficulty and importance of the skull stripping problem has lead to a wide range of tools being developed to tackle it, for example (Brummer et al., 1993; Tsai et al., 1995; Sijbers et al., 1997; Lee et al., 1998; Dale et al., 1999; Lemieux et al., 1999; Hahn and Peitgen, 2000; Huh et al., 2002; Shan et al., 2002; Smith, 2002; Rehm et al., 2004; Rex et al., 2004; Segonne et al., 2004; Zhuang et al., 2006; Chiverton et al., 2007; Sadananthan et al., 2010; Eskildsen et al., 2011; Leung et al., 2011; Galdames et al., 2012), and a number of studies assessing accuracy (Lee et al., 2003; Boesen et al., 2004; Fennema-Notestine et al., 2006; Shattuck et al., 2009).

The similarity in brightness between brain tissue and nearby non-brain tissue, such as dura, makes distinguishing between such tissues on a local level very difficult, if not impossible. Some form of high level knowledge about the structure of these tissues is important in achieving high accuracy. The past few years have seen methods that use large libraries of ground truth data (Eskildsen et al., 2011; Leung et al., 2011), graph cuts (Sadananthan et al., 2010; Dahnke et al., 2011) or increasingly sophisticated combinations of local adaptation and atlas driven refinement (Zhuang et al., 2006; Galdames et al., 2012) to deliver improved accuracy. These methods are increasingly relying on more accurate initialization to deliver the improved accuracy, with typical strategies using combinations of iterative registration to a template or library and inhomogeneity correction. In some cases the results of tissue classification steps are used (Dahnke et al., 2011). These preprocessing stages can be quite complex and computationally intensive, and failure of preprocessing leads to poor scalping results. Any improvement in accuracy is likely to result from a combination of preprocessing characteristics and the refinement step. In addition, it is difficult to make commonly used preprocessing components, such as registration, perform reliably in the presence of non-brain tissue or absence of prior information such as brain landmarks (e.g., via origins set in image headers). These are issues in large studies, where it can become necessary to expend considerable effort selecting preprocessing parameters, or provide some prior information manually.

There are also important tradeoffs between accuracy and reliability to be considered. For example, the graph cut approach introduced in Sadananthan et al. (2010) improved cortical thickness measurement accuracy in FreeSurfer, however, the tendency of the procedure to occasionally remove brain tissue has lead to it being removed as a default option in the FreeSurfer pipeline (Reuter, 2012). Finally there are also subtle differences in definition to consider, such as whether a scalping process is intended to remove bone and scalp, leaving brain and cerebral spinal fluid (CSF), or whether CSF is also removed. These differences certainly influence accuracy scores, but may be less significant in some processing pipelines. For example, inclusion of CSF or bone space (which are dark) is not likely to have a large impact on registration, while inclusion of brighter tissue may. Exclusion of CSF is significant if the final goal is estimation of intra-cranial vault volume. These tradeoffs and design issues need to be considered on a study by study basis and are raised here to highlight the difficulty in providing definitive comparisons between methods.

In this article we revisit the scalping problem using a technique from the field of mathematical morphology that has not been used previously for scalping—the *watershed transform from markers*, combined with aggressive filtering using large kernels. Other forms of the watershed transform have been used in brain scalping (Sijbers et al., 1997; Hahn and Peitgen, 2000; Segonne et al., 2004), but not the very useful marker-based approach. The resulting tool, which we call the *marker based watershed scalper* (MBWSS), is an example of a data-driven approach to segmentation, in which application knowledge is encoded in the sequence of processing steps and choice of parameters, rather than in the form of atlases or shape models derived from training data. This approach is especially useful when ground truth data is not available or expensive to obtain (and may be a useful way of reducing the cost of obtaining the ground truth data by providing reasonable preliminary segmentation results for manual correction), or when the problems is not well suited to model-based approaches.

MBWSS is designed to be applicable to a wide range of data, fast (runtime <30 s), reliable and accurate without the requirement of an expensive preprocessing pipeline. We have constructed the tool using standard computational classes distributed with the *Insight Toolkit* (ITK). MBWSS does not depend on registration or atlases. It is a preprocessing component that is likely to be useful for many analysis procedures.

The MBWSS is an important illustration of the value of constructing segmentation tools using libraries such as ITK. The use of established, optimized, and extensively tested components for filtering and a well described approach to segmentation allowed development of a fast and reliable segmentation tool, without the need for new computational components. The segmentation accuracy of MBWSS, assessed using a publicly accessible resource, is high, illustrating the utility of the morphological approach to segmentation. The fact that the level of accuracy was achieved without resorting to atlases, complex priors or large libraries of examples, can inform development of future segmentation processes.

The flexibility of segmentation using the marker-based watershed transform is further demonstrated by applying the approach to scalping of macaque MR scans. The robustness of the approach is demonstrated using a range of paediatric datasets, including surgical cases.

## 2. Materials and methods

The MBWSS uses a number of techniques that are not well known in the neuroimaging community, namely the morphological watershed transform from markers and a range of filtering operations using large kernels with recursive and separable implementations leading to high efficiency. This section provides a technical introduction to these techniques before describing their application in brain extraction.

### 2.1. Technical background

#### 2.1.1. Morphological watershed transform from markers

The watershed transform is a general purpose tool for image segmentation inspired by the notion of the watershed in geography. A geographical watershed is the line separating two catchment basins—rain falling on one side of the line flows into one catchment basin while rain falling on the other side flows into the second catchment basin. In image segmentation the image brightness forms a surface (commonly called a control surface) that can be segmented into regions by finding watershed lines. The watershed transform has been used in a number of previously reported brain extraction tools (Sijbers et al., 1997; Hahn and Peitgen, 2000). The traditional watershed transform introduced by Beucher and Lantuejoul (1979) is known to be susceptible to over-segmentation—i.e., producing many more regions than desired—and much of the effort in making useful algorithms based on the traditional watershed transform is spent developing pre-processing or post-processing steps to reduce the over-segmentation problem to manageable levels. The best known example in the brain extraction domain is pre-flooding (Hahn and Peitgen, 2000). However, these approaches are less than ideal, often being strongly data dependent or overly complex and difficult to optimize for a particular application.

A general-purpose methodology called the watershed transform from markers (WTM) that avoids over-segmentation was introduced by Meyer and Beucher (1990). WTM has not previously been applied to skull stripping. This approach casts the segmentation process as a series of steps: (a) find markers, (b) create control surface, (c) topology transformation, and (d) watershed transform, with steps (c) and (d) being implemented identically in different applications.

The traditional and marker based watershed transforms are used to segment a control surface (represented by an image) into independent regions by modeling flooding of the surface with fluid. Region boundaries fall on ridge lines in the control surface. It is therefore common to use gradient operators, which transform contrast change (edges) into ridges (lines), in the construction of the control surface. The choice of control surface depends on the nature of the image and the form of regions.

Some algorithms model the process as rain falling on a terrain, but a computationally simpler model, considers water flooding through holes in the surface. In the latter approach each regional minimum corresponds to a hole in the terrain and will produce an independent region in the segmentation. Boundaries of a region occur when flooding regions meet, which happens at ridges in the control surface or at the midpoints of plateaus. These flooding processes can be implemented simply and efficiently with priority queue based methods. By contrast, a rainfall model requires explicit steps to identify and process plateaus or preprocessing steps, such as *lower completion* to eliminate them. Plateaus are a problem for a rainfall model as they trap gradient descent propagation—the virtual raindrop stops on a plateau and cannot reach the minimum of the catchment basin. In general, implementations that support a marker based approach will employ a flooding model.

Noise or natural variations in image intensity will produce additional regional minima, which lead to the classical over-segmentation problem. Reducing the number of regional minima by smoothing or thresholding (pre-flooding) reduces the degree of over segmentation, but neither is a systematic approach.

The watershed transform from markers is a systematic approach that eliminates the over segmentation problem. The basic concept behind WTM is simple—introduce a regional minimum inside each object that needs to be segmented, apply a transformation to the control surface that eliminates other regional minima, eliminate plateaus, and apply a watershed transform to the resulting surface. The first two steps are collectively referred to as imposing regional minima while eliminating plateaus is known as lower completion. The imposed minima are known as markers. The advantage of this approach is that one region is produced per minimum; so precise control over the number of regions is possible. In addition the segmented regions are guaranteed, by construction, to contain the markers. The markers, which are typically represented by a label image, are the *a priori* knowledge about the segmentation task in the form of approximate object position and number of objects.

**Figure 1:**
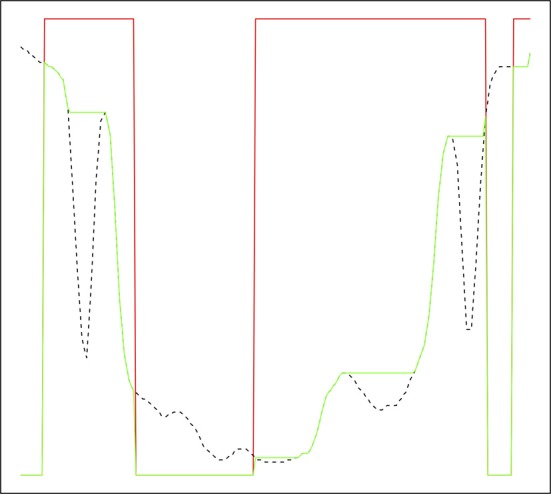
**Reconstruction by erosion of 1D signal from markers**. Original signal in dashed black, marker signal in red, reconstructed signal in green. The green signal only has regional minima where the marker signal is zero.

It is possible to explicitly impose regional minima by setting locations in the control image corresponding to marker voxels to the minimum possible value and carrying out a reconstruction by erosion or recursive conditional erosion. This process is illustrated in Figure 1 for a one-dimensional signal, where the original signal is transformed to have regional minima only at markers. A number of more common watershed algorithms, particularly those based on priority queues (Beucher and Meyer, 1992; Meyer, 1994), are able to avoid the need to perform the preprocessing steps of imposition of minima and lower completion, via the nature of the flooding operation. This leads to efficient implementations of the marker approach. A traditional watershed transform can be computed using a marker-based watershed algorithm by using regional minima as markers. Both Meyer's and Beucher's algorithms are available in the ITKv4 *itk::MorphologicalWatershedFromMarkersImageFilter* class (Beare and Lehmann, 2006). The difference between the two algorithms is that Meyer's marks the watershed line between regions with a single voxel thick line or surface (in 3D) while Beucher's algorithm produces touching regions.

The WTM is a very stable algorithm and is parameter free—the user does not need to set stopping conditions or parameters controlling the flooding process. Both WTM and traditional watershed are greedy algorithms in which a class is assigned to every voxel, resulting in an algorithm that stops when all voxels are assigned to a class. The flooding process, which models water flooding a terrain, is governed entirely by relative values of voxels and topology in the control image. It is informative to compare these characteristics to those of the level-set family of methods (Sethian, 1999), which is currently popular. Level sets are heavily dependent on image pre-filtering and terms describing energy functions and lack well defined stopping conditions. Thus WTM approaches can be developed much more easily and perform more reliably than level set approaches in many circumstances. The potential advantages of the level set family of methods in some applications are that it is not necessary to define the number of regions and they have some ability to cope with broken boundaries.

#### 2.1.2. Efficient filtering using large kernels

Most common image filtering operations replace an image voxel with some function of voxels in a kernel around the voxel. The execution time of direct implementations of such filters is therefore strongly dependent on the number of voxels in the kernel—in three-dimensional images a doubling of kernel dimensions (from *n* × *n* × *n* to 2*n* × 2*n* × 2*n*) will lead to an approximately eightfold increase in the execution time. This cost has traditionally restricted users to small kernels, such as 3 × 3 × 3 voxels.

However, a number of approximations and/or optimizations exist for a range of useful filter types that reduce the complexity from *O*(*n*^*k*^), where *k* is the number of dimensions, to *O*(const). Examples include separable and recursive algorithms for Gaussian convolution (Deriche, 1990; Lindeberg, 1991) and grayscale erosions and dilations (Van Herk, 1992; Adams, 1993; Gil and Werman, 1993). Separable methods decompose a k dimensional kernel into a cascade of k, one-dimensional operations, thereby reducing complexity to *kO*(*n*). Recursive operations exploit redundancy in the computation to further reduce the complexity, often to constant time. A simple example of a recursive algorithm is computation of the running mean along a line by updating a sum—the sum can be updated by adding the incoming value and subtracting the outgoing value and the mean computed by dividing by the length of the kernel—requiring three operations, no matter what the kernel size. Separable filters are also relatively simple to implement in parallel, leading to higher performance on multi-core CPUs. In this application we use gaussian smoothing operations, large grayscale morphology operations (erosions/dilations) with 3D rectangular structuring elements, and binary morphology using spherical structuring elements. Another option for improving the execution time relative to direct implementation of large kernels is repeated application of “unit” kernels, leading to complexity *k*O(const). A limited range of kernel shapes and filtering functions can be decomposed this way and performance is not as high as optimized approaches.

The grayscale morphology classes offered by ITKv4—*itk::GrayscaleDilateImageFilter, itk::GrayscaleErodeImageFilter*, *itk::GrayscaleMorphologicalClosingImageFilter*, *itk::GrayscaleMorphologicalOpeningImageFilter*—offer a range of algorithms. Morphology using arbitarily shaped, flat, structuring elements can be performed relatively efficiently using a sliding histogram approach, which reduces complexity from *O*(*n*^*k*^) to approximately *O*(*n*^*k* − 1^). Morphology using structuring elements with a restricted range of shapes—rectangles or polygons in 2D or boxes in 3D—that can be composed using a cascade of line structuring elements can be performed using recursive algorithms described by Van Herk (1992) and Gil and Werman (1993). These classes offer filtering times independent of structuring element size for these shapes.

Binary morphology using spherical structuring elements is offered by a series of ITKv4 classes available via the *Insight Journal* (Beare, 2008a,b; Beare and Jackway, 2011). These classes use operations based on parabolic structuring functions (van den Boomgaard et al., 1996). Parabolic structuring functions are separable and a number of efficient algorithms exist for the 1D case (van den Boomgaard et al., 1996; Felzenszwalb and Huttenlocher, 2004). These functions can be used to compute binary erosions/dilations by exact discs or spheres efficiently.

Gaussian smoothing, another workhorse filtering operation, is also separable and efficient approximations via digital filters are available for the 1D case. ITKv4 offers a series of classes for fast linear filtering using the methods described by Deriche (1990) and Lindeberg (1991). These classes include *itk::SmoothingRecursiveGaussianImageFilter*, *itk::GradientMagnitudeRecursiveGaussianImageFilter*, *itk::DiscreteGaussianImageFilter*, and *itk::DiscreteGaussianDerivativeImageFilter*.

The difference in execution time between an efficient, specialized filter and a general purpose, direct implementation can be dramatic, with an operation taking an infeasibly long time to execute dropping to a small component of the total execution time. This has an enormous impact on design of image filtering algorithms. For example, consider two types of morphological filtering operations applied to a typical 1 mm resolution brain scan (258 × 258 × 182 voxels)—grayscale dilations using cubic structuring elements and binary dilations using spherical structuring elements. A cubic structuring element can be implemented directly, using a brute force algorithm, a more efficient sliding histogram, by repeated dilations using a unit cube structuring element or by decomposition into orthogonal line structuring elements. The options for a spherical structuring element include direct, sliding histogram or via parabolic structuring functions in the case of binary images (there is no unit sphere available for the repeated applications). Execution times for a range of sizes and tools are illustrated in Table 1. In typical cases we see a speedup of approximately two orders of magnitude over direct implementations using ITKv4 for moderately sized cubic structuring elements. Speedup over a widely used, general purpose, neuroimaging arithmetic tool, fslmaths, is a factor of approximately 500 for grayscale operations. Timing tests performed using an Intel(R) Core(TM) i7 CPU 920, 2.67 GHz, 4 core hyperthreaded, running Ubuntu Linux. Other timing measures in this paper use the same host.

**Table 1:** **Execution times, in seconds, for morphological dilations using a variety of structuring elements and tools with different specialized implementations**.

	**GS cube 3**	**GS cube 11**	**Bin cube 3**	**Bin cube 11**	**Bin sphere radius 11**
Fslmaths—direct	17	534	15	533	2528
Fslmaths—repeated unit	17	78	15	73	
ITKv4—direct	1.1	87	1.1	87	65
ITKv4—histogram	1.7	18	1.6	18	15
ITKv4—vanHerk/GilWerman	0.917	0.9	0.9	0.9	
ITKv4—parabolic					0.55
ITKv4—parabolic (four threads)					0.15

*GS and Bin refer to grayscale and binary images. Gaps in the table correspond to operations that cannot be performed with some tools. Advantages of selecting the appropriate specialized algorithm for large kernels is significant. The disparity between general and specialized methods increases with structuring element size*.

#### 2.1.3. Morphology on connected components

Binary images produced by thresholding and possibly subsequent morphological filtering operations can be further filtered based on the size and shape of connected components. ITKv4 provides an extensive framework for shape-based morphology on binary images (Lehmann, 2007). The *itk::BinaryShapeOpeningImageFilter* and *itk::BinaryShapeKeepNObjectsImageFilter* are used in this application. The first allows connected components to be retained based on a number of useful attributes, with volume being a widely used example. The second allows the *n* objects with the highest (or lowest) attribute (such as volume) to be retained.

#### 2.1.4. Histogram-based threshold estimation

One of the most widely used tools in image segmentation is thresholding, with thresholds determined via analysis of gray-level histograms. A wide variety of techniques have been developed for different histogram characteristics. ITKv4 provides classes implementing the more popular methods (Beare, 2011). These classes also offer the facility to estimate the threshold in a region defined by a mask. This application uses the method introduced by Otsu (1979).

### 2.2. Brain extraction algorithm for human scans

**Figure 2:**
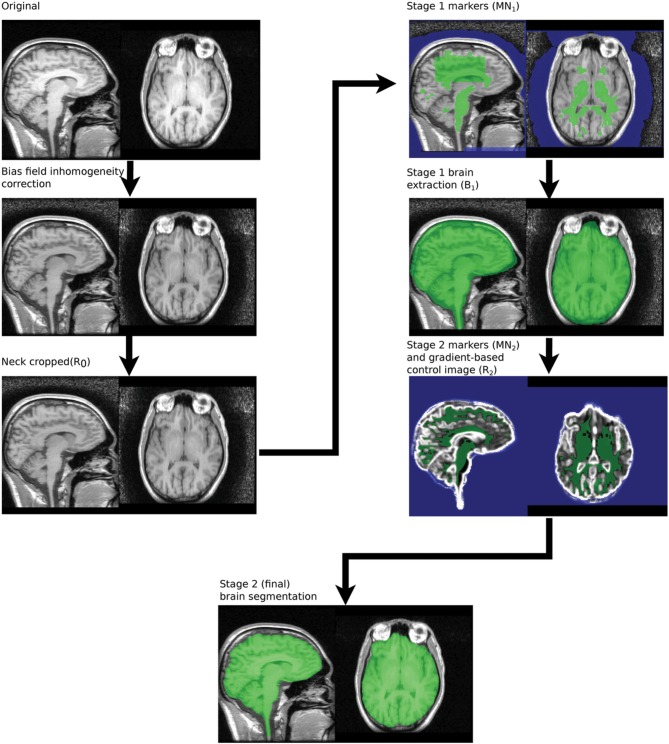
**Summary of processing steps used in MBWSS**.

The MBWSS is a multistage process consisting of Stage 1—fast preprocessing, marker generation, and watershed segmentation—and Stage 2—marker refinement and a second watershed segmentation using a different control surface. The basic strategy for each stage is to use a pair of markers—one inside the brain and one outside—and to apply a WTM using these markers and an appropriate control surface. Stage one uses the inverted T1 as the control image, as was originally proposed in Hahn and Peitgen (2000). Stage 2 uses a control surface derived from the image gradient. This algorithmic structure transforms the segmentation problem into one of finding markers and developing the appropriate control surface, and is an example of the classical morphological segmentation approach described by Meyer and Beucher (1990). The process is illustrated in Figure 2. We now describe these steps in detail.

#### 2.2.1. Bias field inhomogeneity correction

Strong bias fields can influence many processing steps. However, high quality bias correction is relatively slow and is best performed either as part of the tissue classification process or with the aid of a brain mask. The MBWSS is not particularly sensitive to bias field inhomogeneities, but correction of strong inhomogeneities using very simple and fast methods can improve reliability on low quality images. A method similar to that of Haselgrove and Prammer (1986), in which a bias field is estimated using low spatial frequency components has been developed using ITKv4 filtering framework. Specifically the *itk::BoxMeanImageFilter* is used to apply a large (radius = 30 mm) smoothing kernel, thus retaining only the low spatial frequency components. This is a rough estimate of the inhomogeneity field that provides sufficient correction for the MBWSS to function reliably in the presence of quite strong brightness inhomogeneity. This step is optional.

#### 2.2.2. Neck cropping

The field of view used in human imaging studies can vary considerably. In many cases a significant length of neck is included, which leads to large volumes of bright, non-brain tissue being visible. Presence of this tissue can make location of the brain using simple methods more difficult. For example, the center of gravity of the image, which is frequently used to provide an initialization for brain extraction and tissue classification (Smith, 2002; Glaser, 2011), may not fall inside the brain.

Neck cropping is implemented as follows:

1. Threshold the input image using Otsu's method (Otsu, 1979) and keep the largest connected component.
2. Determine the superior-most slice of the largest connected component.
3. Blank all slices more than 180 mm inferior to the superior-most slice. No slices are blanked if the inferior-most image slice is closer than 180 mm to the superior-most slice of the largest connected component.
4. The center of mass of the most superior 35 mm of the largest connected component is computed. This position, COM_top_ is used in the marker generation process.

The size parameter used in neck cropping is deliberately conservative to avoid cropping brain tissue if the head is imaged at an unusual angle, but can be reduced for paediatric cohorts. The neck cropping step is not optional, as the parameter COM_top_ is required by subsequent steps. However, the step has no effect on images that do not require it—i.e., when the bottom of the image is less than 180 mm from the superior-most slice. Only a few slices are blanked is the example show in Figure 2.

#### 2.2.3. Stage 1 marker generation

**Figure 3:**
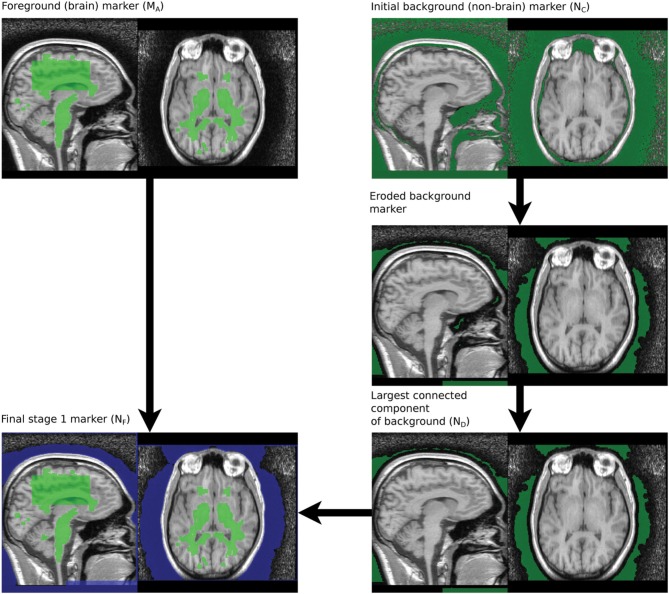
**Processing steps for generating Stage 1 markers**.

The markers provide starting regions for the watershed transform and must not cross the boundaries of regions that are to be segmented separately—i.e., the marker for the brain must fall entirely within the brain while the non-brain marker must fall entirely outside the brain. The process described below uses aggressive morphological filtering with large structuring elements to achieve this. In the following notation images denoted *R*_*n*_ are grayscale and derived from the original T1, with *R*_0_ referring to the neck-cropped T1. Images denoted *M*_*n*_ and *N*_*n*_ are masks of various forms derived from thresholded and filtered versions of the original T1. *N* is used to denote masks of non-brain tissue. The term “merge” refers to combining images using a voxel-wise maximum operator. The process to produce the brain marker is:

1. A box, with sides length 40 mm is created 50 mm below COM_top_.
2. The 50% brightness level of the T1 image, *R*_0_, in the box is computed, *T*1_med_.
3. A mask of T1 voxels between *T*1_med_ and 1.25*T*1_med_ is created, a morphological opening by a sphere radius 2 mm applied and the connected components touching the box retained, to produce *M*_*A*_, Figure 3.

The marker for the non-brain tissue is created as follows and also illustrated in Figure 3:

1. The brain marker (*M*_*A*_) is inverted and the resulting mask eroded by a large sphere (radius = 10 mm) to produce *N*_*A*_.
2. *N*_*A*_ is smoothed using an opening by large spherical structuring element (radius = 30 mm) and the largest connected component retained, to produce *N*_*B*_.
3. *R*_0_ is filtered using a morphological opening using 5 mm cubic structuring element to produce *R*_*A*_.
4. The Otsu threshold for the area defined by *N*_*B*_ is computed for the *R*_*A*_ image. The previous filtering step reduces the number of possible classes and improves the reliability of Otsu thresholding.
5. Voxels defined as bright by this threshold are removed from *N*_*B*_ to produce *N*_*C*_.
6. *N*_*C*_ is eroded using a spherical structuring element (radius = 5 mm) and the largest connected component retained to produce *N*_*D*_.
7. *N*_*D*_ is dilated by a spherical structuring (radius = 6 mm, slightly larger than the erosion in Step 6), to produce *N*_*E*_.
8. Any slices blanked during neck cropping are filled in *N*_*E*_ to produce *N*_*F*_.

*N*_*F*_ is a mask with the background marker overlapping the scalp by a small margin.

The brain and background markers are merged to create a single image (*MN*_1_, Figure 3), with voxels in the brain marker assigned a value of 1, those in the background marker assigned a value of 2 and non-marker voxels assigned a value of 0.

#### 2.2.4. Stage 1 brain extraction using the watershed transform from markers

**Figure 4:**
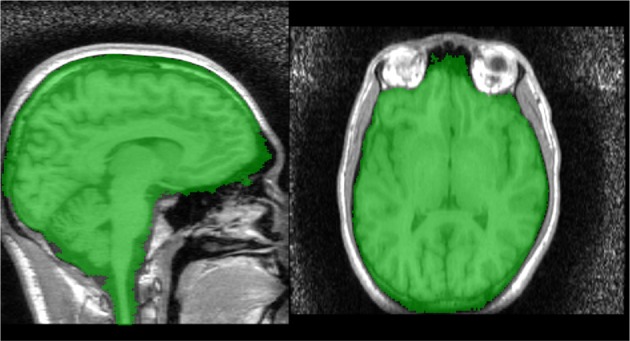
**Stage 1 segmentation result**.

The marker image, *MN*_1_, created using the procedure described in section 2.2.3 is used in conjunction with a control image to produce an initial brain/non-brain segmentation using a watershed transform from markers. The control image is the inverted T1. As discussed in section 2.1.1, the watershed transform segments regions such that the region boundaries fall along bright ridge lines. Bright ridge lines in the inverted T1 image correspond to the bone and CSF between the brain and scalp. Given the control and marker images, the watershed transform from markers is parameter free and requires no stopping conditions. The brain mask, *B*_1_ illustrated in Figure 4, produced by this first stage segmentation process is created by selecting those voxels in the watershed output with a value of 1 (the brain label). The brain mask is intended to be a conservative one, including all brain tissue while clipping none, at the cost of including some non-brain tissue.

#### 2.2.5. Stage 2 marker generation

For some applications it is useful to refine the Stage 1 segmentation results further. This is especially useful when dural layers or cancellous bone (marrow) are included in the segmentation. We approach the problem by using a new control image and a more detailed marker generation process. These methods rely on the first stage segmentation to define a region in which the new markers will be placed—within a small distance (10 mm) of the Stage 1 boundary. The marker generation for Stage 2 produces small non-brain markers in very bright or very dark areas close to the Stage 1 boundary.

A new brain marker is created as follows:

1. Compute the median, *t*_50_, of *R*_0_ in the region defined by *B*_1_.
2. Create a border zone, *B*_*bz*1_, of *B*_1_ by subtracting an eroded version (*B*_1*e*_) of *B*_1_ from the original. A border zone with 10 mm thickness is appropriate, meaning *B*_1*e*_ is created by eroding *B*_1_ using a spherical structuring element with 10 mm radius.
3. Bright voxels that are definitely brain tissue are selected to create a new brain maker: *M*_2_ = (*R*_0_ ≥ *t*_50_) * *B*_1*e*_.

The process for computing markers for dark areas is:

1. Apply a small (1 mm) erosion to the T1 scan to produce *R*_*A*_. This removes fine structure typical of dural layers.
2. Mask the eroded *R*_*A*_ with *B*_1_, to produce *R*_*B*_.
3. Compute a large, masked mean filter to estimate the local brightness of the raw T1 scan. A masked mean filter ignores voxels outside the mask when computing the mean. *B*_1_ is the mask and a 30 × 30 × 30 mm kernel is used to produce the local brightness image, *R*_*lb*_.
4. An image adjusted for local brightness is computed: *R*_ladj_ = *R*_*B*_/*R*_*lb*_.
5. Dark voxels are defined as those less than 60% of the local mean brightness. Those within the border zone are retained to produce a marker image: *N*_dark_ = (*R*_ladj_ < 0.6) × *B*_*bz*1_.

Very bright T1 signal produced by cancellous bone (marrow) does not occur in all scans, but are not uncommon. If such signal is present it is likely to be included within the Stage 1 mask. It is therefore useful to produce markers on regions corresponding to such signal to indicate that they should be segmented as background in Stage 2. The process for producing bright markers is:

1. Compute the median, *t*_50_, of *R*_0_ in the region defined by *M*_2_.
2. Create a border zone, *B*_*bz*2_, of *B*_1_ by subtracting an eroded version (*B*_2*e*_) of *B*_1_ from the original. This border zone is 1/2 the thickness of *B*_*bz*1_ and is created by eroding with a spherical structuring element radius 3.3 mm.
3. Compute the bounding box of *B*_1*e*_, in order to approximately define the superior-inferior extent of the brain. Superior brain regions are defined as those 90 mm or more above the inferior extent of the bounding box. A mask defining this zone, *B*_sup_, is created.
4. A mask of bright voxels is created by thresholding and masking by the border zone: *N*_bright_ = (*R*_0_ > 1.25 * *t*_50_) × *B*_*bz*2_ × *B*_sup_.

Dark and light markers are merged and connected components smaller than 10 mm^3^ discarded, to produce a new set of border zone markers, *N*_*bz*_. The Stage 1 segmentation of non-brain tissue is merged with *N*_*bz*_ to create the final background marker, *N*_2_.

**Figure 5:**
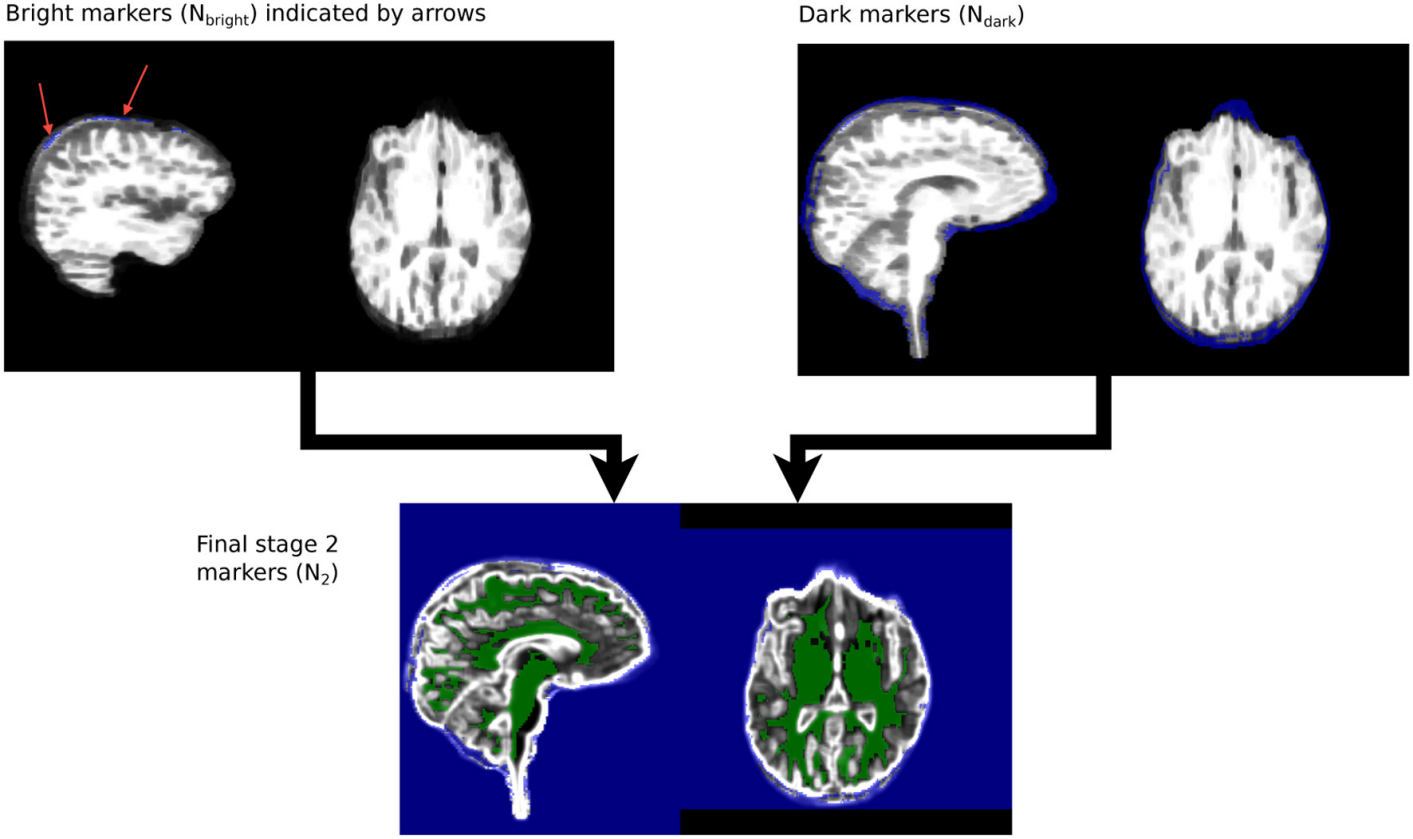
**Stage 2 marker generation phases**. *N*_dark_ and *N*_bright_ are overlaid on *R*_*B*_. *N*_2_, the final stage 2 marker image, is overlaid on *R*_*D*_.

*M*_2_ and *N*_2_ are merged to create a single marker image, *MN*_2_ for Stage 2 segmentation. The steps are illustrated in Figure 5.

#### 2.2.6. Stage 2 control image generation

The new control image is derived from a combination of the T1 gradient and the raw T1 values. The latter is necessary as not all brain boundaries correspond to regions with high gradients. This strategy ensures that boundaries that are already correct after Stage 1 are retained. The control surface is generated as follows:

1. Set all voxels in *R*_*B*_ brighter than *T*_50_ to *T*_50_, to produce *R*_*C*_. This reduces the strength of white matter-gray matter transitions and has been used previously in Smith (2002).
2. The border voxels of the Stage 1 segmentation are extracted from *R*_*C*_, and the median intensity subtracted, to produce *R*_*D*_. Bright voxels in this image correspond to boundaries in the Stage 1 segmentation that fall on brighter tissue, such as the cranial nerves and vessels in the vicinity of the brain stem.
3. A smoothed morphological gradient image, *R*_*E*_, is computed using convolution with a Gaussian kernel. A kernel with a 1 mm standard deviation is used. A morphological gradient operation is used as it is easy to deal with mask boundaries.
4. The final control image, *R*_2_ is created using a voxel-wise maximum of *R*_*D*_ and *R*_*E*_: *R*_2_ = max(*R*_*D*_, *R*_*E*_) (Figure 5).

#### 2.2.7. Stage 2 brain extraction using the watershed transform from markers

**Figure 6:**
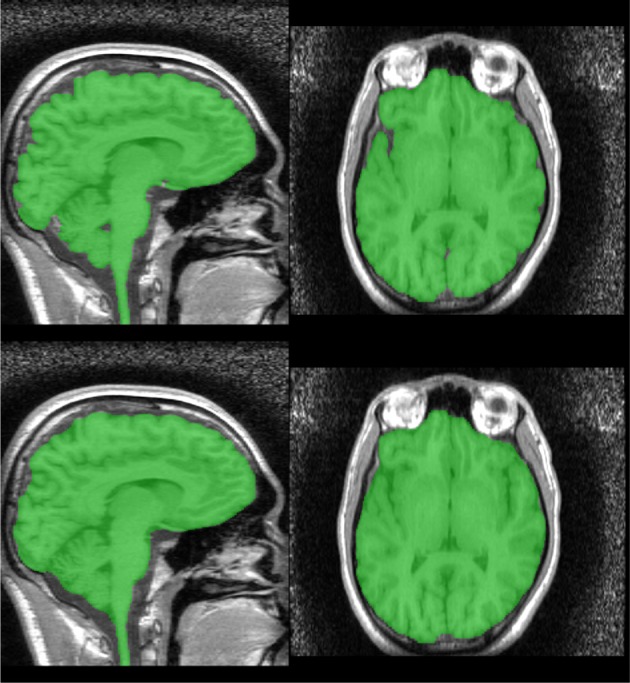
**Segmentation produced by Stage 2—raw (top) and smoothed (bottom)**.

A second watershed transform from markers is applied using *MN*_2_ as the marker image and *R*_2_ as the control. The resulting brain segmentation is dilated by 1 mm, to account for the erosion applied to create *R*_*A*_ in Step 1, and voxels from the bright marker image, *N*_bright_ are removed, as the dilation may result in these voxels being included (Figure 6).

#### 2.2.8. Mask smoothing

The resulting mask may be smoothed using morphological opening and closings with spherical structuring elements. We smooth the Stage 1 segmentation using a opening (radius = 5 mm) followed by a closing (radius = 6.5 mm) and the Stage 2 segmentation using a closing (radius = 6.5 mm) (Figure 6).

### 2.3. Brain extraction for macaque scans

Macaque skulls and brains have different geometry to humans, with large muscles attached to the skull and smaller brains, and the MRI scans have different characteristics, notably much stronger brightness inhomogeneity. This leads to changes in the marker generation details, although the basic strategy of creating markers for brain and non-brain tissue is the same. In this case we use knowledge of approximate brain size more directly, rather than via filtering operations, as the similarity in dimensions of the macaque brain to some of the surrounding muscles makes selection via kernel filter size unreliable. This approach is informative, and illustrates an alternative mechanism to including prior information.

Notation for images is as previously described.

#### 2.3.1. Bias correction

Brightness inhomogeneity can be very severe in macaque scans. The simple bias correction procedure outlined in section 2.2.1 is used to reduce severity.

#### 2.3.2. Neck cropping

The process is almost identical to that described in section 2.2.2, with the most obvious difference being the distance below the top of the skull that blanking starts. A value of 80 mm is used for macaques instead of the 180 mm used for humans.

In addition, COM_top_ is computed using the most superior 15 mm, rather than 35 mm.

#### 2.3.3. Stage 1 marker generation

**Figure 7:**
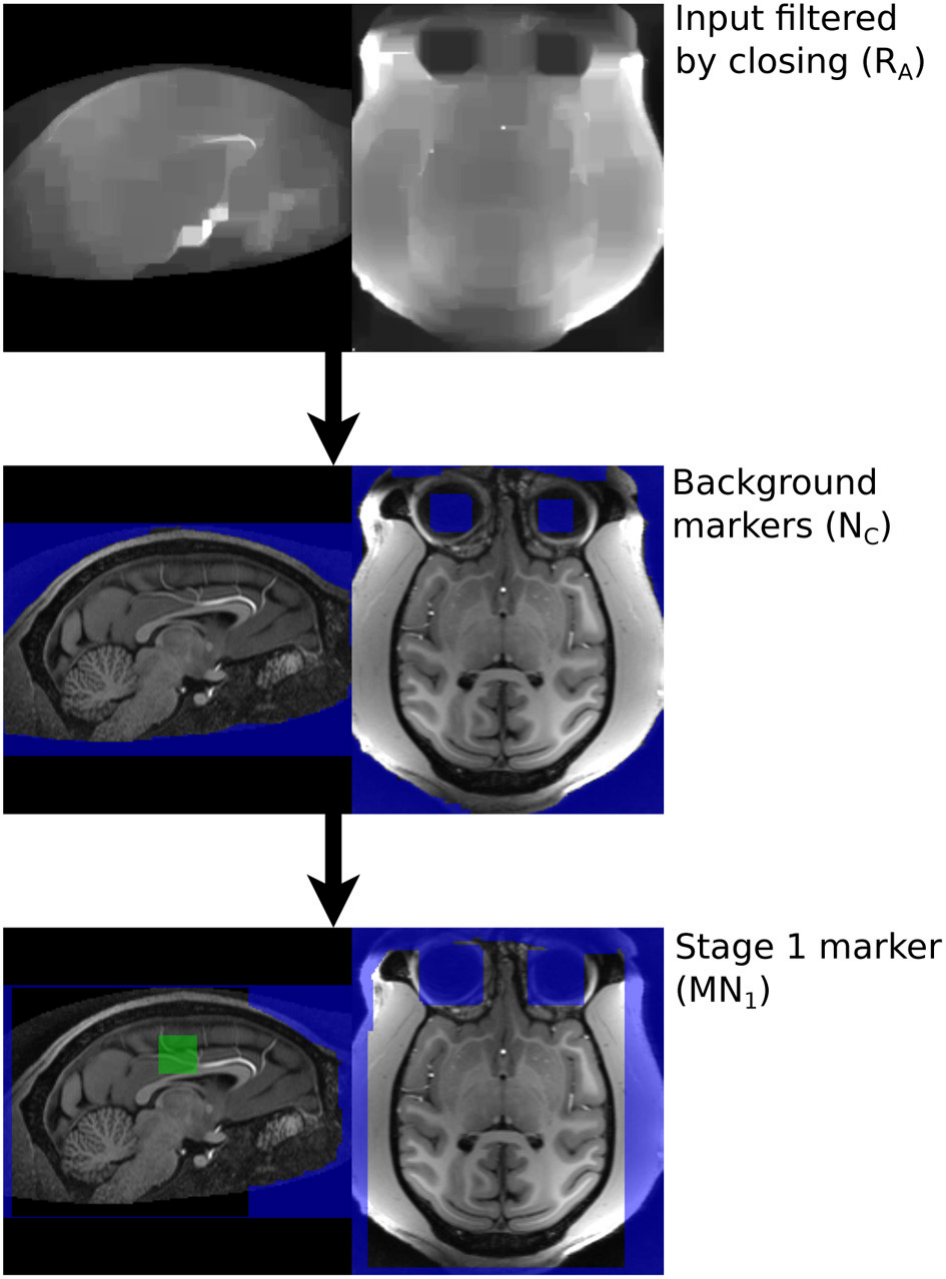
**Processing steps producing stage 1 marker, *MN*_1_**.

The marker for non-brain tissue involves simple geometric operations and filtering to locate markers in structures such as eyes, and is generated as follows:

1. Initial marker for non-brain tissue, *N*_*A*_ includes the entire image, filled with label 2.
2. A box, size (left → right) × (anterior → posterior) × (superior → inferior) = 70 mm × 90 mm × 65 mm, centered at 35 mm below COM_top_, is blanked, to produce *N*_*B*_.
3. A grayscale closing, using rectangular box structuring element, 8 × 8 × 8 mm is applied to create *R*_*A*_. This removes narrow dark regions such as the brain-scalp space, while retaining large dark regions (Figure 7).
4. The 1% quantile of *R*_*A*_ is computed to define a mask of voxels above background intensity, *B*_*A*_.
5. The 25% quantile of voxels in mask *B*_*A*_ in *R*_*A*_ is used as a threshold to create a binary image *B*_*B*_, which selects dark regions in *R*_*A*_. The dark regions include the interior of large spaces such as eyes (which remain dark following the closing in Step 3).
6. *B*_*B*_ is combined with the inverse of *B*_*A*_ to create *B*_*C*_ (Figure 7).
7. The region in *B*_*C*_, extending from posteriorly from 20 mm anterior to COM_top_, is blanked. This restricts new markers generated following the closing to dark anterior spaces, namely the eyes.
8. Connected components in *B*_*C*_ smaller than 100 mm^3^ are discarded and the result dilated by 3 mm to produce *B*_*D*_.
9. *B*_*D*_ and *N*_*B*_ are merged to form *N*_*C*_ (Figure 7).

This produces a roughly boxed brain, with no background marker in the brain, and markers in large, dark, anterior regions, such as the eyes.

The brain marker is generated as follows:

1. A box, size 10 mm × 10 mm × 15 mm, centered at 35 mm below COM_top_, in *N*_*C*_, is filled with label 1, to produce a final marker image, *MN*_1_.

#### 2.3.4. Stage 1 brain extraction using the watershed transform from markers

**Figure 8:**
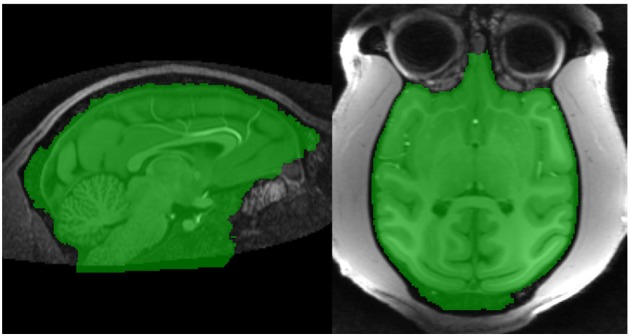
**Stage 1 segmentation for macaque**.

This stage is identical to the Stage 1 segmentation step described in section 2.2.4, with a watershed transform from markers, using an inverted T1 image as the control image and *MN*_1_ as the marker. The Stage 1 segmentation is generated by selecting watershed output voxels with value of 1 (Figure 8). This is also a conservative segmentation. However, the inferior boundaries are often very inaccurate due to the severity of brightness inhomogeneity, and correcting this drives stage 2.

#### 2.3.5. Stage 2 marker generation

The segmentation of non-brain tissue produced by Stage 1, *N*_*D*_ is used as the non-brain marker for Stage 2. The new brain marker is constructed from the Stage 1 brain segmentation, *M*_*B*_ as follows:

1. *M*_*B*_ is eroded using a spherical structuring element, radius 5 mm, to produce *B*_*E*_.
2. The median, *t*_50_, of the raw T1 in the region defined by *M*_*B*_ is computed.
3. A new brain marker, *M*_*C*_, corresponding to bright parts of the Stage 1 segmentation, is created: *M*_*C*_ = (*R*_*A*_ > *t*_50_) × *B*_*E*_.

**Figure 9:**
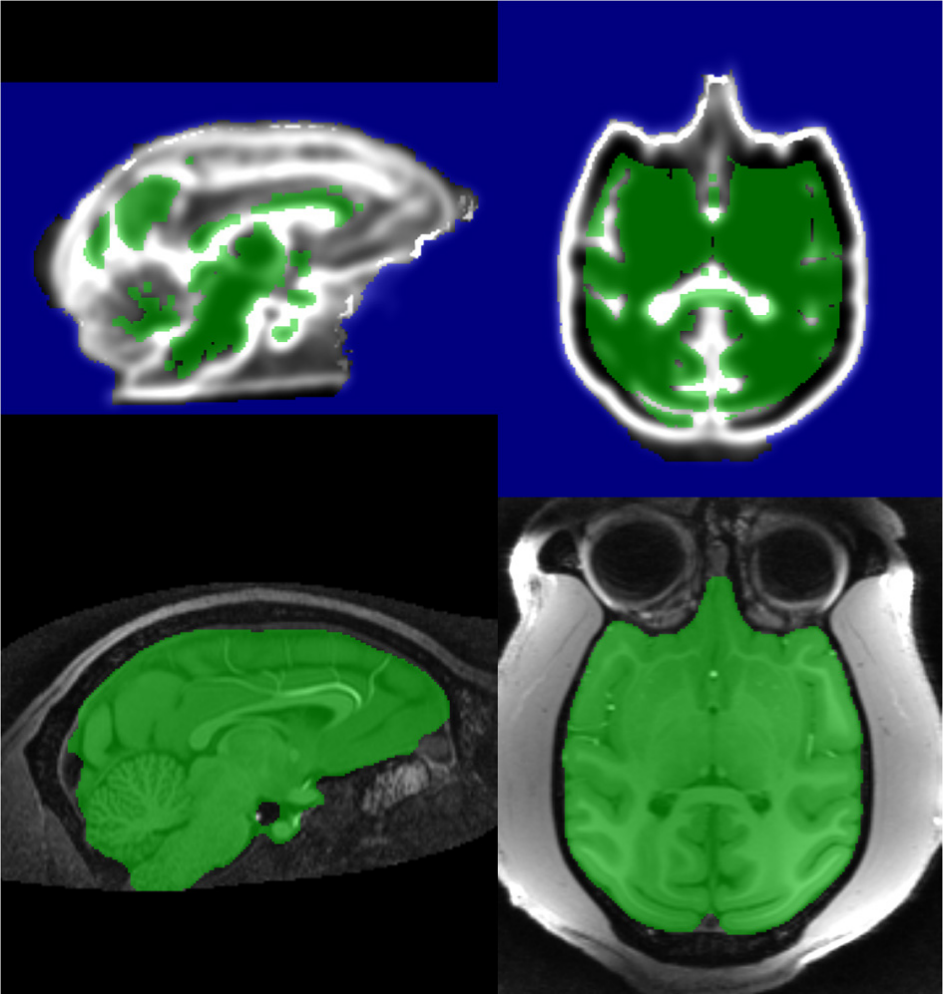
**Stage 2 marker for macaque on gradient control image (top) and resulting stage 2 segmentation (bottom)**.

*M*_*C*_ and *N*_*D*_ are merged to create *MN*_2_, the Stage 2 marker (Figure 9). This procedure is simpler than Stage 2 for humans as we are doing less fine tuning of markers.

#### 2.3.6. Stage 2 control image

The control image is constructed using the method described in section 2.2.6, to produce *R*_2_, without the initial erosion applied in Step 1 as there are fewer fine dural structures (Figure 9).

#### 2.3.7. Stage 2 brain extraction using the watershed transform from markers

A second watershed transform from markers is applied using *MN*_2_ as the marker image and *R*_2_ as the control. There is no additional dilation step as the control image was not eroded (Figure 9).

### 2.4. Validation

#### 2.4.1. Accuracy

Accuracy of the method described above for human brains was assessed using the publicly accessible *Segmentation Validation Engine* (SVE) (Shattuck et al., 2009). This service provides 40 raw T1 scans and allows segmentation results to be uploaded and compared to a manually generated ground truth using Dice, Jaccard, sensitivity and specificity scores. An additional final process step was applied to clip the brain stem segmentation prior to upload to the SVE, as the MBWSS typically segments a portion of brain stem while other methods do not. The MNI brain mask was registered to the segmentation mask using FLIRT (Jenkinson and Smith., 2001) and the inferior face of the MNI space used to clip the brain stem.

Brain masks created from five macaque scans were compared with manually created brain masks intended for registration purposes using Dice and Jaccard scores. The masks were constructed by manually editing the output of BET applied to macaque scans. The boundaries of these manual masks were not particularly accurate, especially in dark areas, as they were intended to remove tissue that would interfere with registration.

#### 2.4.2. Robustness

MBWSS is designed to be a fast, first stage extraction tool run at the beginning of a processing pipeline, with the goal of making subsequent steps simpler and more accurate. It is useful to consider how robust the approach is. Robust in this context refers to the frequency of “significant” errors, with the precise definition of significant being dependent on the specific processing pipeline. We compared brain masks generated by MBWSS to those generated by more computationally intensive tools in a paediatric cohort and an adolescent cohort. The paediatric cohort consisted of 29 participants randomly selected from the Victorian Infant Brain Study (VIBeS), mean age 7.57 ± 0.21 while the adolescent cohort consisted of 14 participants from 2 year followup in a traumatic brain injury study, mean age 13.25 ± 1.95. The VIBeS cohort is 85% very preterm (≤ 30 weeks gestation). All scans performed on a 3T Siemens Trio. Brain masks were created using MBWSS, FreeSurfer version 5.3.0's preprocessing pipeline (autorecon1), the graph cut method in VBM8 (Dahnke et al., 2011), standard BET (Smith, 2002), BET with robust brain center estimation (BET -R) and BET with bias field and neck cleanup (BET -S). Dice coefficients and false positive and false negative rates were computed to compare VBM8's graph-cut method to all other methods.

Finally, the application to intra-surgical scans was tested using two cases scanned on a 3T high field movable intra-operative MR Siemens scanner (IMRIS, Manitoba, Canada) placed in an operating room with radiofrequency shielding.

## 3. Results

Segmentation accuracy scores from the Segmentation Validation Engine and macaque datasets are listed in Table 2. Scores for the robustness tests sre listed in Tables 3, 4.

**Table 2:** **MBWSS segmentation accuracy measures from SVE and macaque datasets**.

	**Jaccard**	**Dice**	**Sensitivity**	**Specificity**
SVE	0.9436 ± 0.0057	0.9710 ± 0.0030	0.9662 ± 0.0081	0.9957 ± 0.0014
Macaque	0.86 ± 0.018	0.92 ± 0.011	0.88 ± 0.015	0.994 ± 0.004

**Table 3:** **Dice coefficients, mean false positive and false negative and worst false positive and false negative scores for all methods compared to graph-cut for the paediatric cohort**.

	**Paediatric**				
	**Dice**	**Mean false Pos**	**Mean false Neg**	**Worst false Pos**	**Worst false Neg**
MBWSS	0.962 ± 0.005	0.02 ± 0.008	0.05 ± 0.011	0.04	0.09
FreeSurfer	0.964 ± 0.005	0.064 ± 0.012	0.006 ± 0.003	0.09	0.01
BET	0.943 ± 0.002	0.03 ± 0.03	0.079 ± 0.020	0.13	0.14
Robust BET	0.964 ± 0.0055	0.010 ± 0.007	0.06 ± 0.010	0.031	0.0867
BC NC BET	0.934 ± 0.023	0.0025 ± 0.002	0.12 ± 0.039	0.012	0.252

*BC NC BET refers to BET with bias correction and neck cleanup*.

**Table 4:** **Dice coefficients, mean false positive and false negative and worst false positive and false negative scores for all methods compared to graph-cut for the adolescent cohort**.

	**Adolescent**				
	**Dice**	**Mean false Pos**	**Mean false Neg**	**Worst false Pos**	**Worst false Neg**
MBWSS	0.958±0.004	0.021 ± 0.011	0.06 ± 0.011	0.05	0.09
FreeSurfer	0.953±0.010	0.086 ± 0.020	0.004 ± 0.003	0.01	0.11
BET	0.720±0.096	0.41 ± 0.14	0.044 ± 0.013	0.5	0.06
Robust BET	0.738 ± 0.123	0.379 ± 0.183	0.055 ± 0.021	0.54	0.086
BC NC BET	0.960 ± 0.012	0.013 ± 0.004	0.064 ± 0.024	0.023	0.135

*BC NC BET refers to BET with bias correction and neck cleanup*.

### 3.1. Intra-surgical examples

**Figure 10:**
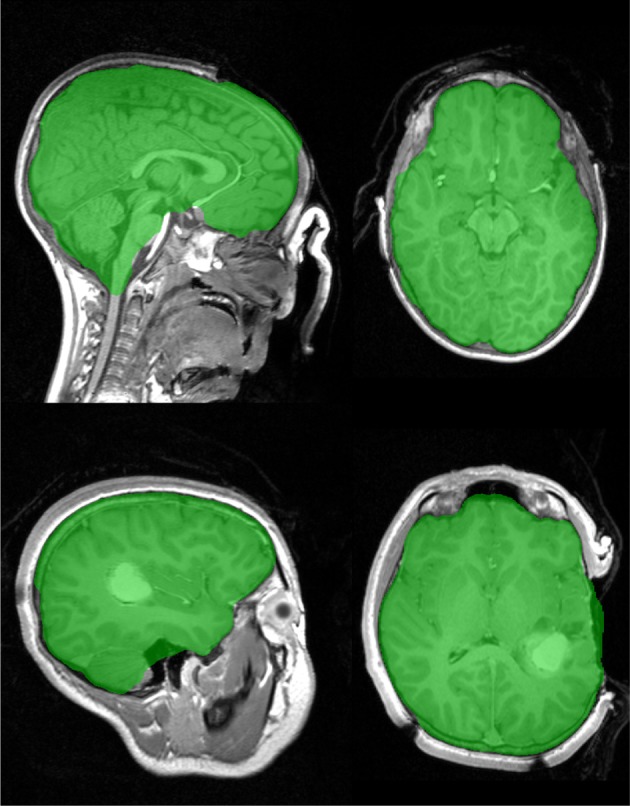
**Brain extraction (Stage 1), for two intra-surgical scans with open skull**. Top image shows a contrast enhancing tumor. Acquisition on IMRIS surgical scanner.

**Figure 11:**
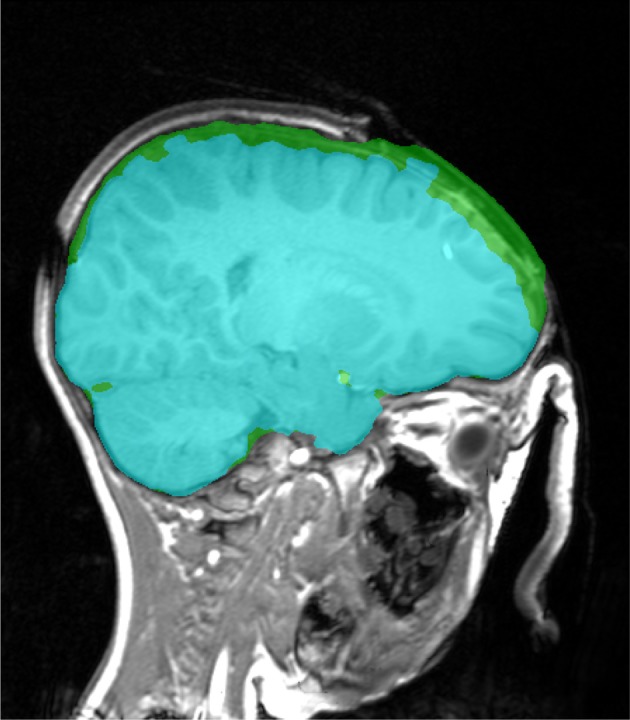
**Brain extraction (Stage 1, in green) and corresponding graph cut segmentation (in cyan), which has a incorrectly removed brain tissue in the vicinity of the open skull**.

MR acquisition during surgery is possible with in-theatre scanners. The examples in Figure 10 have open skulls and contrast agents have been injected. An open skull breaks one of the assumptions of the Stage 1 marker generation process, so a minor modification (reducing the size of dilation in Step 7 in section 2.2.3) is necessary to prevent brain tissue being included in the background marker, resulting in a less than ideal marker generation process and a larger amount of non-brain tissue being included in the final segmentation. Despite this the brain extraction results are useful for further analysis. An example of failures observed with graph cut is illustrated in Figure 11.

### 3.2. Execution time

Execution time for the different methods is summarized in Table 5. The MBWSS execution time includes 2.4 s for bias correction, 9.6 s for stage 1 marker generation and segmentation and 7 s for stage 2 marker generation and segmentation

**Table 5:** **Execution times for methods on 256 × 124 × 256 images provided by the SVE, and a 170 × 190 × 127 macaque scan**.

	**Human scan**	**Macaque scan**
MBWSS	19	11
FreeSurfer	2640	
VBM8 graph cut	420	
BET	3.4	
Robust BET	12	
BC NC BET	495	

## 4. Discussion

### 4.1. Segmentation performance

At the time of writing MBWSS was at position 21 on the SVE table, with eight different methods ahead by generally small margins. In fact, the top 85 entries (note that there are many repeats of the same methods in this list) have Dice scores of 0.96 or better, with the top method scoring 0.981. By this metric, many methods are doing very well. It is therefore informative to consider what the differences in scores mean—the Dice similarity coefficient between a brain mask and a single voxel dilation of the same mask is 0.959. This is lower than the mean scores produced by the top 85 entries on the SVE website. Differences between all of these methods is therefore likely to be very subtle.

The top performing methods, such as BEaST (Eskildsen et al., 2011), MAPS (Leung et al., 2011), gain high performance based on combinations of ground truth training data, careful preprocessing and registration techniques. Others, such as the graph cut method in VBM8 (Dahnke et al., 2011) employ an initial tissue classification and bias correction step in addition to careful noise filtering.

It is interesting that MBWSS—a data driven segmentation method, built using standard, general purpose, image segmentation and filtering components—is able to perform at a level close to the state of the art. It is especially interesting that MBWSS functions with only the most basic preprocessing—optional and very cheap bias correction and no registration to any form of template. It is therefore an ideal starting point for many refinement approaches that may be able to function more efficiently if a reasonably scalped brain is available.

Application to other cohorts—paediatric, adolescent and intra surgical scans—illustrates the flexibility and of the approach. MBWSS showed quite close correspondence with the graph cut method. The highest level of disagreement between the two methods occurred in scans with atrophied brains, in which the graph cut method included more dark (non-brain) voxels, leading to a high false negative scores. The difference between the methods in these cases was unlikely to be significant for most applications.

FreeSurfer's performance on the two cohorts illustrates a different design approach—the segmentation is deliberately conservative, with a very low false negative rate, but moderate false positive rate. The segmentations produced by FreeSurfer are quite similar to those produced by the first stage of MBWSS, which is not surprising considering the use of watershed transform steps in both.

BET, in three forms, had variable performance on the test cohorts. Standard BET had poor mean Dice coefficients for the adolescent cohort. BET with robust brain centering performed well on the paediatric cohort and poorly on the adolescent cohort. BET with bias correction and neck cleanup performed well on the adolescent cohort but not as well on the paediatric cohort. In all cases the masks with the highest rates of false negative voxels had obvious regions of misclassified brain voxels.

Illustrations of the segmentations produced by the methods are provided in supplementary material.

### 4.2. Segmentation performance—macaques

The similarity coefficients for macaque brain masks were lower than those achieved for human scans, however, the difference was largely due to the lower quality manual segmentation results available. The manual masks were generated purely for the purposes of registration and included large areas of dark voxels.

Illustrations of the macaque manual segmentations are provided in supplementary material.

### 4.3. Parameter selection

MBWSS has a lot of parameters which could, in principle, be individually tuned. All results for human scans in this paper, apart from the surgical cases, were obtained using the same set of default parameters. Surgical cases required one preprocessing step to be modified, as discussed. The macaque scans were also processed with identical parameters.

Tuning is possible and can be targeted based on the steps that fail. For example, a poor stage 1 segmentation may be caused by incorrect marker generation, which suggests tuning parameters relating to that part of the process.

### 4.4. Segmentation error types

The most common form of error observed in MBWSS results was inclusion of non-brain tissue in the form of thick cancellous bone. In some cases the tissue has very similar contrast to brain tissue, so there is minimal gradient between the brain and non-brain tissue. This is a situation where a data driven approach is typically likely to perform badly, as higher level knowledge is necessary to correctly solve the segmentation problem.

### 4.5. Execution time

The execution time for MBWSS was less than 20 s for all steps on the SVE images. Standard BET and BET with robust brain center estimation are the only faster methods, while BET with bias correction and neck cleanup is much slower (8 min). Standard BET is rarely used due to the high rates of poor segmentation. The graph cut method in VBM requires initial tissue classification and image smoothing and takes 7 min. FreeSurfer, which uses an iterative approach involving registration, takes 43 min. It is not always meaningful to compare execution times directly—some of the methods discussed here were designed to take advantage of tools that were already available, or are part of processing pipelines that produce data used later for other purposes. However, it is interesting to observe that MBWSS does achieve the relatively low execution time while offering good reliability, yet it has not been optimized for speed. The execution time was achieved by using specialized components that were available in the ITKv4 library. In many cases these filters implement state of the art algorithms and are able to take advantage of multi-core CPUs. MBWSS is therefore an example of what can be achieved via the good software engineering practice of resusable software components. Significant further improvements in speed would be possible by careful use of cropping to reduce image size (current neck cropping only blanks regions, rather than removing them).

### 4.6. Source code availability

The source code implementing the MBWSS algorithm for humans and macaques is freely available from Beare (2013). There are two, command line based, applications which have been tested under various linux distributions and Apple OSX. Build instructions are included with the source code.

## 5. Conclusion

This article has described the application of a classical morphological segmentation approach to the problem of brain extraction in human and macaque MRI scans. The methods for both human and macaque scans are very similar and are entirely data driven. The methods do not employ computationally intensive preprocessing steps and are intended to be used early in processing analysis procedures. The use of established software components from the ITK facilitated the development of a high speed and efficient tool.

### Conflict of interest statement

The authors declare that the research was conducted in the absence of any commercial or financial relationships that could be construed as a potential conflict of interest.
