# Supplementary material for: Brain extraction using the watershed transform from markers
Source: Front Neuroinform. 2013 Dec 9;7:32. doi: 10.3389/fninf.2013.00032 (PMC3856384; doi:10.3389/fninf.2013.00032)
Supplement: Supplementary file 1 [file Presentation1.PDF]

Frontiers in Neuroinformatics 1November 2013

Supplementary Material - Brain extraction using the watershed transform from markers

Richard Beare [1,2,*], Jian Chen [1,2], Christopher L Adamson [1], Timothy Silk [1], Deanne K Thompson [1,4,5], Joseph Y M Yang [1,6], Vicki A Anderson [3], Marc L Seal [1], and Amanda G Wood [7]

[1] *Developmental Imaging, Murdoch Childrens Research Institute, Melbourne, Victoria, Australia*
[2] *Stroke and Aging Research Group, Department of Medicine, Southern Clinical School, Monash University, Melbourne, Victoria, Australia*
[3] *Clinical Sciences, Murdoch Childrens Research Institute, Melbourne, Victoria, Australia*
[4] *Victorian Infant Brain Studies, Murdoch Childrens Research Institute, Melbourne, Victoria, Australia*
[5] *Florey Dept of Neuroscience and Mental Health, University of Melbourne, Victoria, Australia*
[6] *Department of Neurosurgery, Royal Childrens Hospital, Melbourne, Victoria*
[7] *School of Psychology, University of Birmingham, Edgbaston, United Kingdom*

Correspondence*:
Richard Beare
Developmental Imaging, Murdoch Childrens Research Institute, Royal Children's Hospital, Melbourne, Victoria, Australia, 3052, Richard.Beare@mcri.edu.au

Research Topic

1 INTRODUCTION

This section contains segmentations illustrating behaviour of various brain segmentation methods applied to an adolescent and a paediatric cohort. The methods and corresponding color codings are:

- VBM 8 graph cuts (used as the ground truth in the paper). Light green.
- Marker based watershed scalper (MBWSS), stage 1. Purple.
- Marker based watershed scalper (MBWSS), stage 2. Dark Green.
- FreeSurfer (autorecon1). Orange.
- BET. Dark blue.
- BET with robust brain centering (BET -R). Light blue.
- BET with bias field correction and neck cleanup (BET -B). Pink.

In addition, illustrations of manual segmentations of macaque scans, used for accuracy measures, and the corresponding automated segmentations are provided.

2 ILLUSTRATIONS OF PAEDIATRIC COHORT SEGMENTATION RESULTS

The examples for which MBWSS performed most poorly, compared to graph cut, in terms of false positives and false negatives are illustrated in Figures 1 and 2. More typical examples are show in Figures 3 and 4.

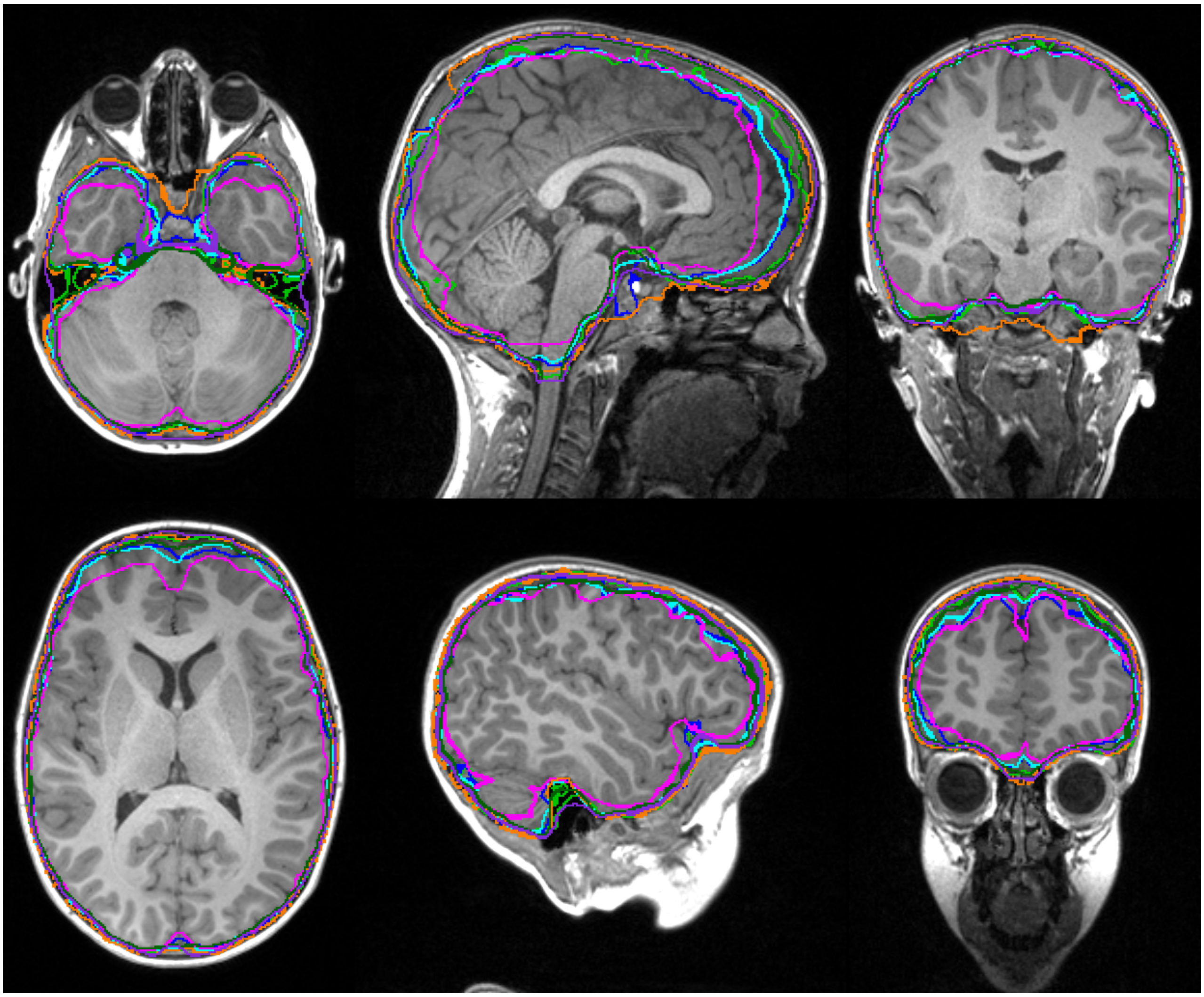

Figure 1. Paediatric cohort - MBWSS worst false positive score. VBM 8 graph cuts, MBWSS stage 1,MBWSS stage 2, FreeSurfer, BET, BET -R, BET -B.

3 ILLUSTRATIONS OF ADOLESCENT COHORT SEGMENTATION RESULTS

The examples for which MBWSS performed most poorly, compared to gcut, in terms of false positives and false negatives are illustrated in Figures 5 and 6. More typical examples are show in Figures 7 and 8. Poor false negative scores have occurred when the subject has enlarged ventricles and the graph cut result is away from the brain edge. High false positive scores occur when graph cut has incorrectly clipped some brain.

4 MANUAL MACAQUE SEGMENTATIONS

Examples of brain extraction for macaque scans are illustrated in Figures 9 to 11. Color coding for segmentation of macaque scans is:

- Light green - manual segmentation for registration purposes.
- Dark blue - MBWSS, stage 2.

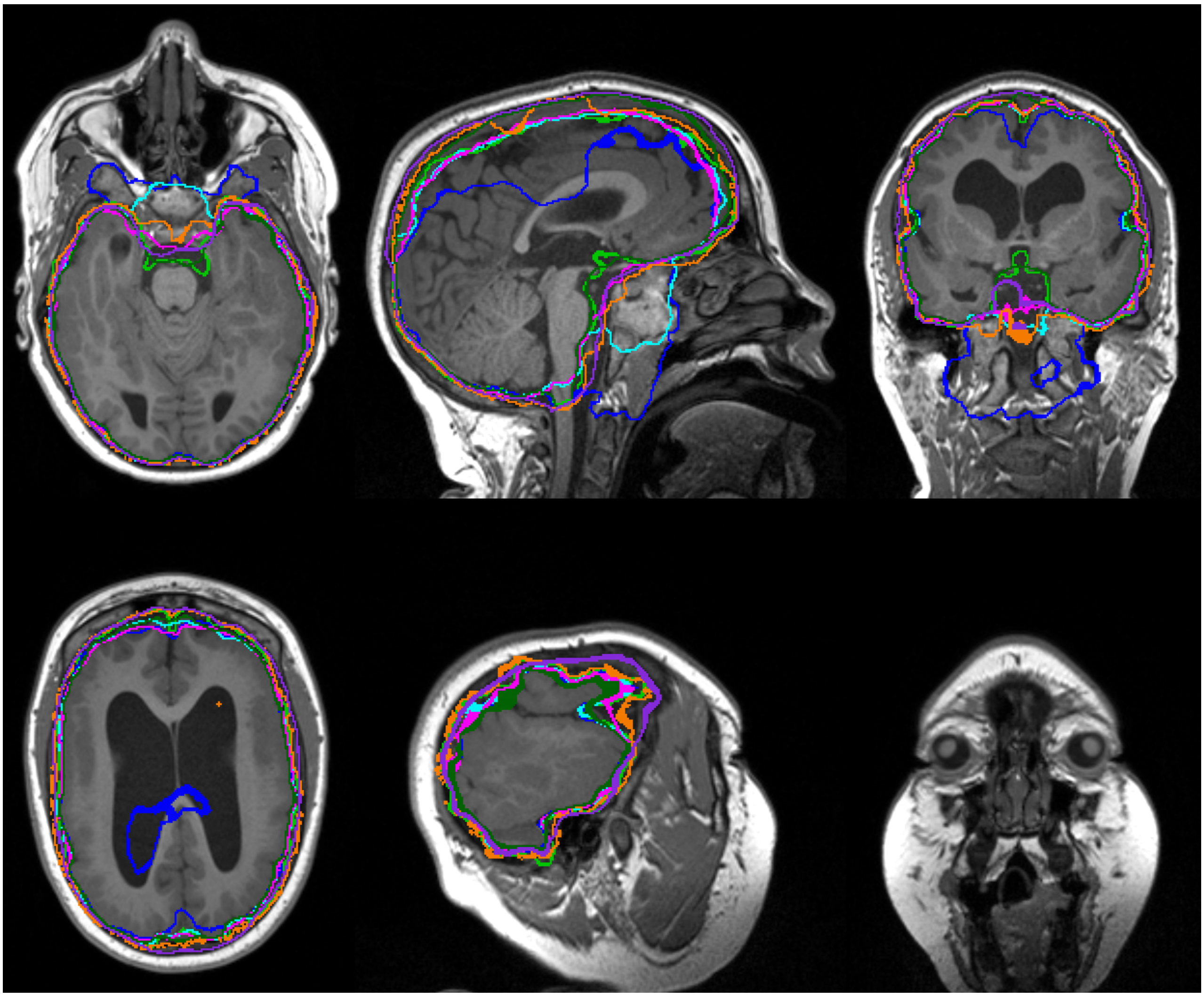

Figure 2. Paediatric cohort - MBWSS worst false negative score. VBM 8 graph cuts, MBWSS stage 1,MBWSS stage 2, FreeSurfer, BET, BET -R, BET -B.

The manual segmentations were generated for registration purposes, and are clearly not aligned with the brain edges. This contributed to the relatively low accuracy scores achieved for macaque scans. Note the extreme brightness inhomogeneity in these scans.

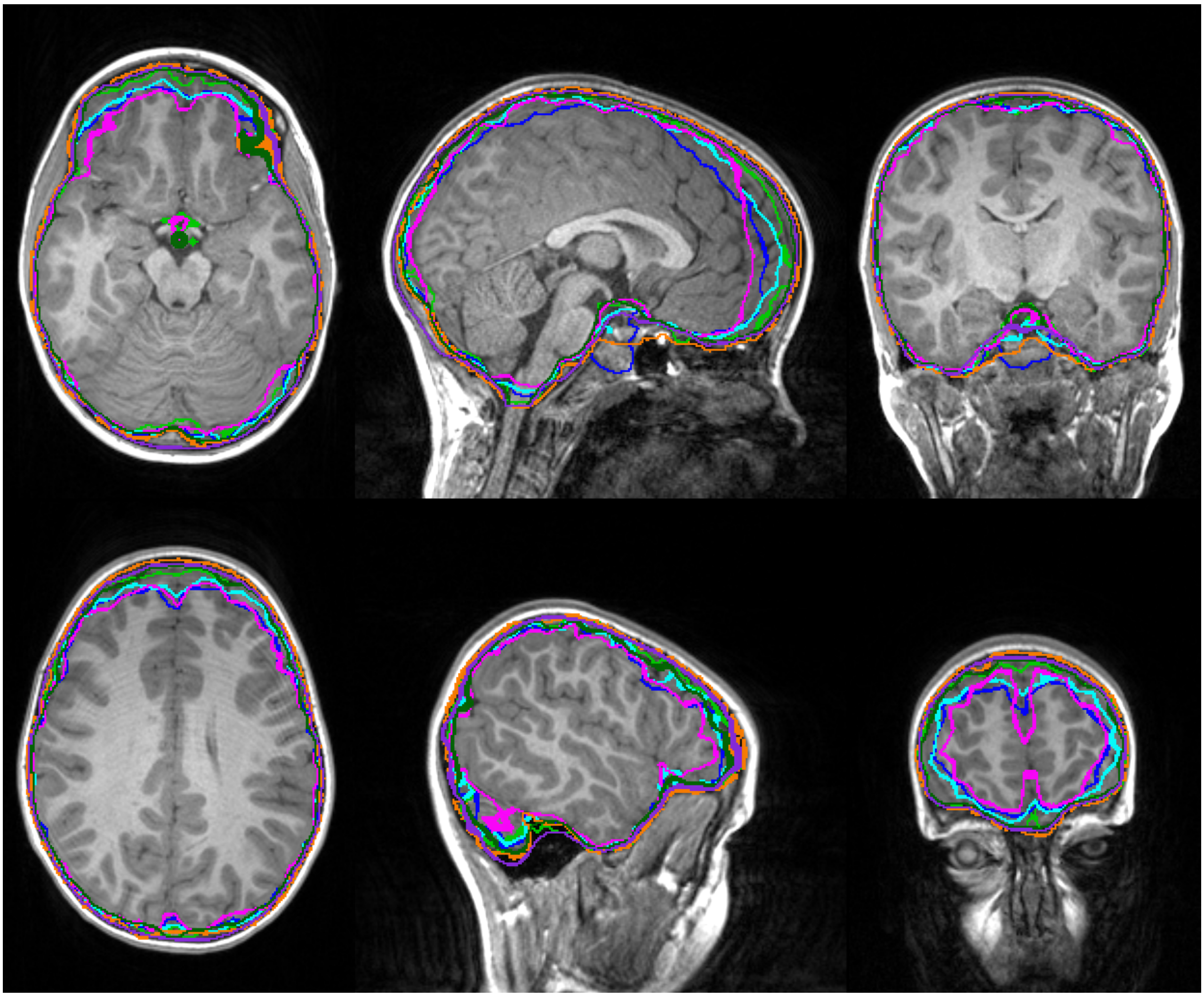

Figure 3. Paediatric cohort - typical example (median false positive score from MBWSS). VBM 8 graph cuts, MBWSS stage 1,MBWSS stage 2, FreeSurfer, BET, BET -R, BET -B.

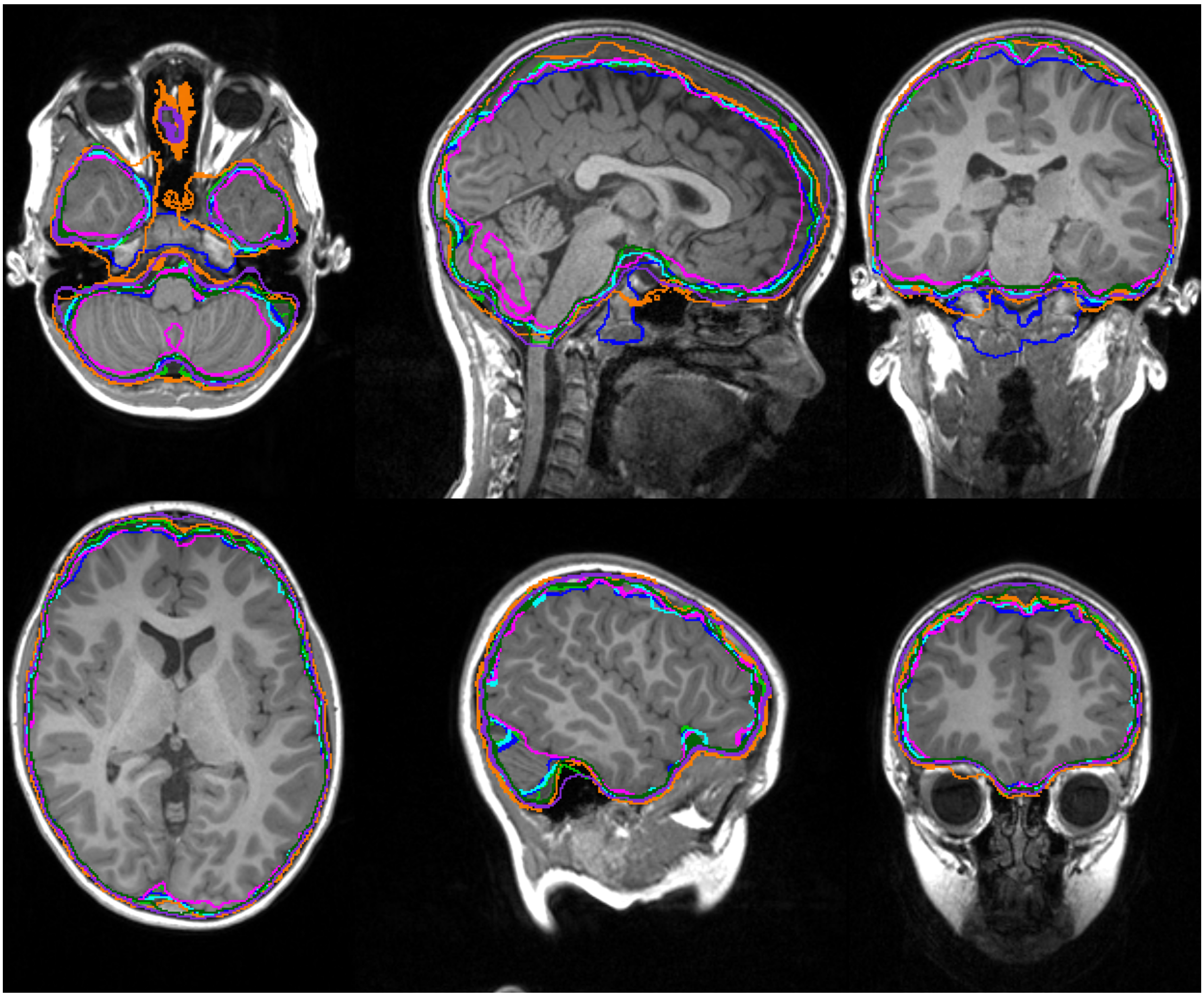

Figure 4. Paediatric cohort - typical example (median false negative score from MBWSS). VBM 8 graph cuts, MBWSS stage 1,MBWSS stage 2, FreeSurfer, BET, BET -R, BET -B.

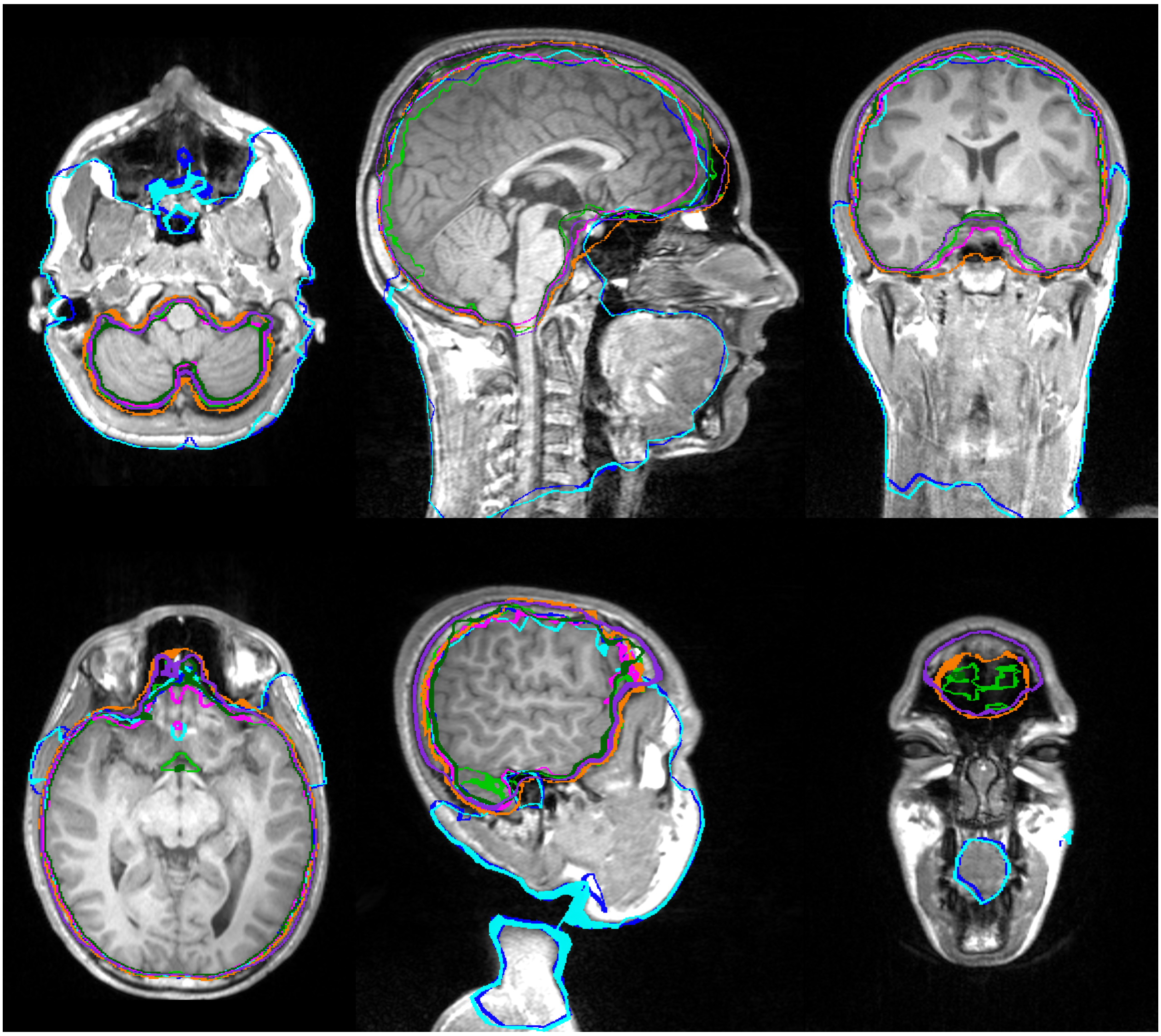

Figure 5. Adolescent cohort - MBWSS worst false positive score. VBM 8 graph cuts, MBWSS stage 1,MBWSS stage 2, FreeSurfer, BET, BET -R, BET -B.

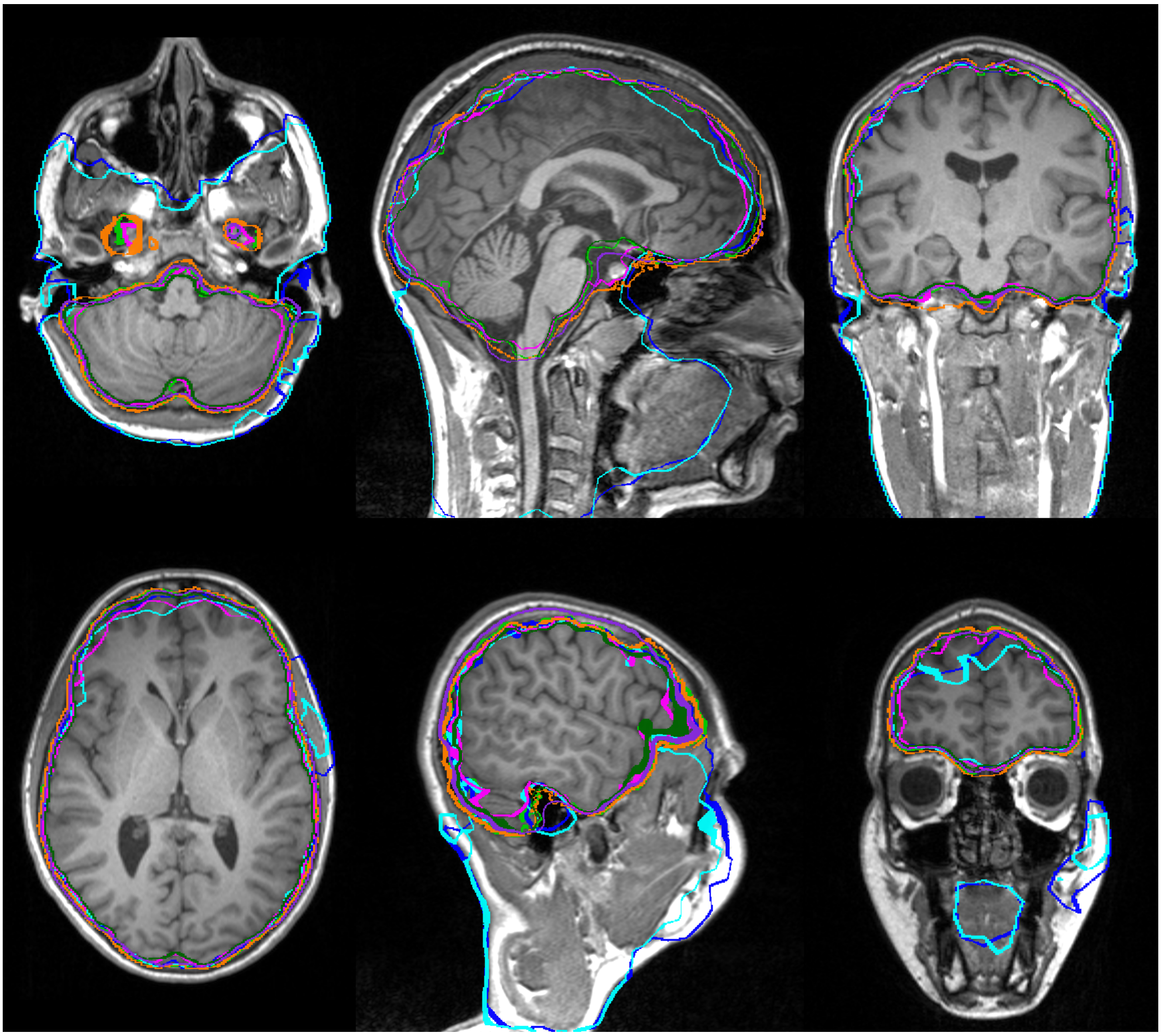

Figure 6. Adolescent cohort - MBWSS worst false negative score. VBM 8 graph cuts, MBWSS stage 1,MBWSS stage 2, FreeSurfer, BET, BET -R, BET -B.

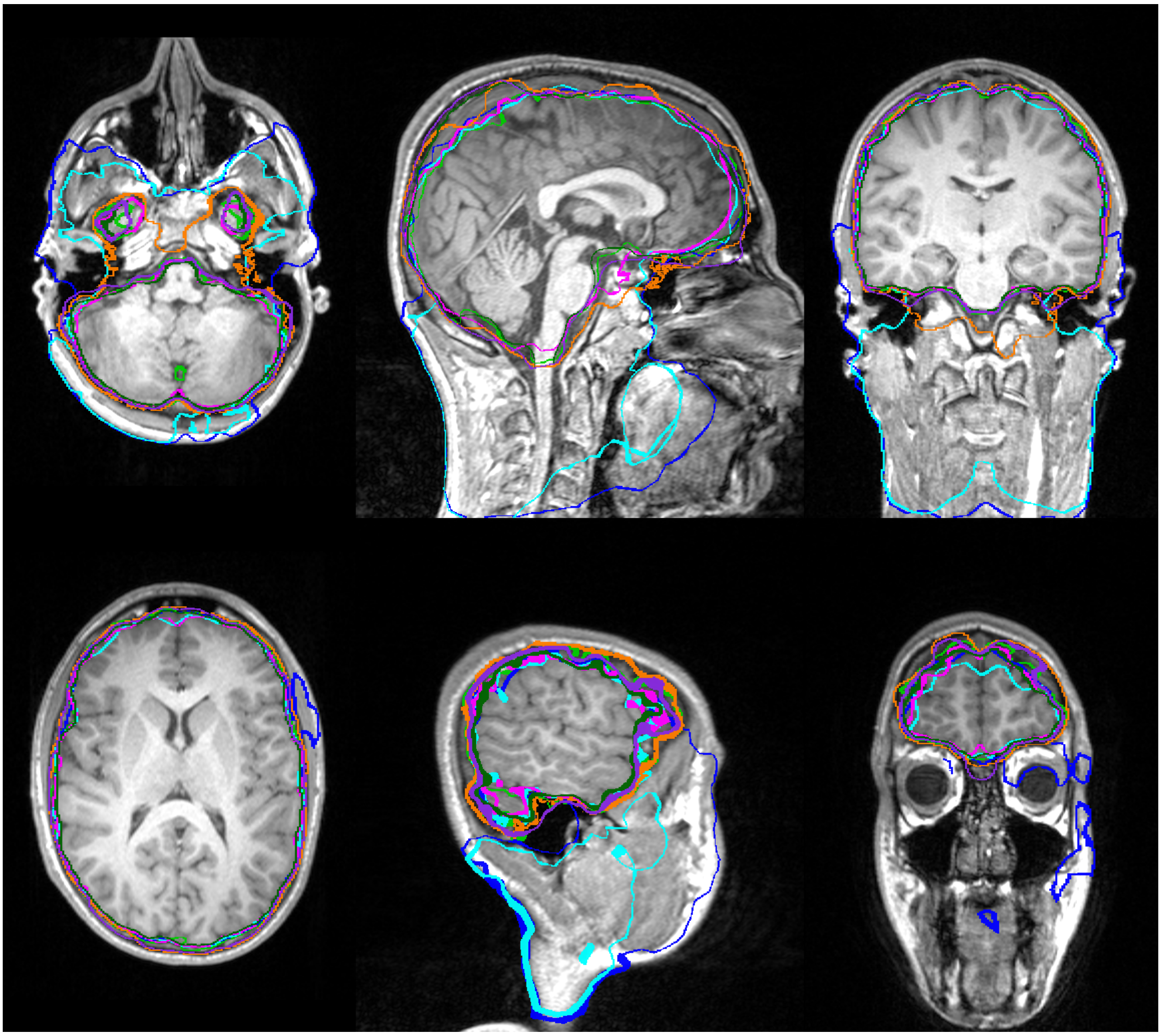

Figure 7. Adolescent cohort - typical example (median false positive score from MBWSS). VBM 8 graph cuts, MBWSS stage 1,MBWSS stage 2, FreeSurfer, BET, BET -R, BET -B.

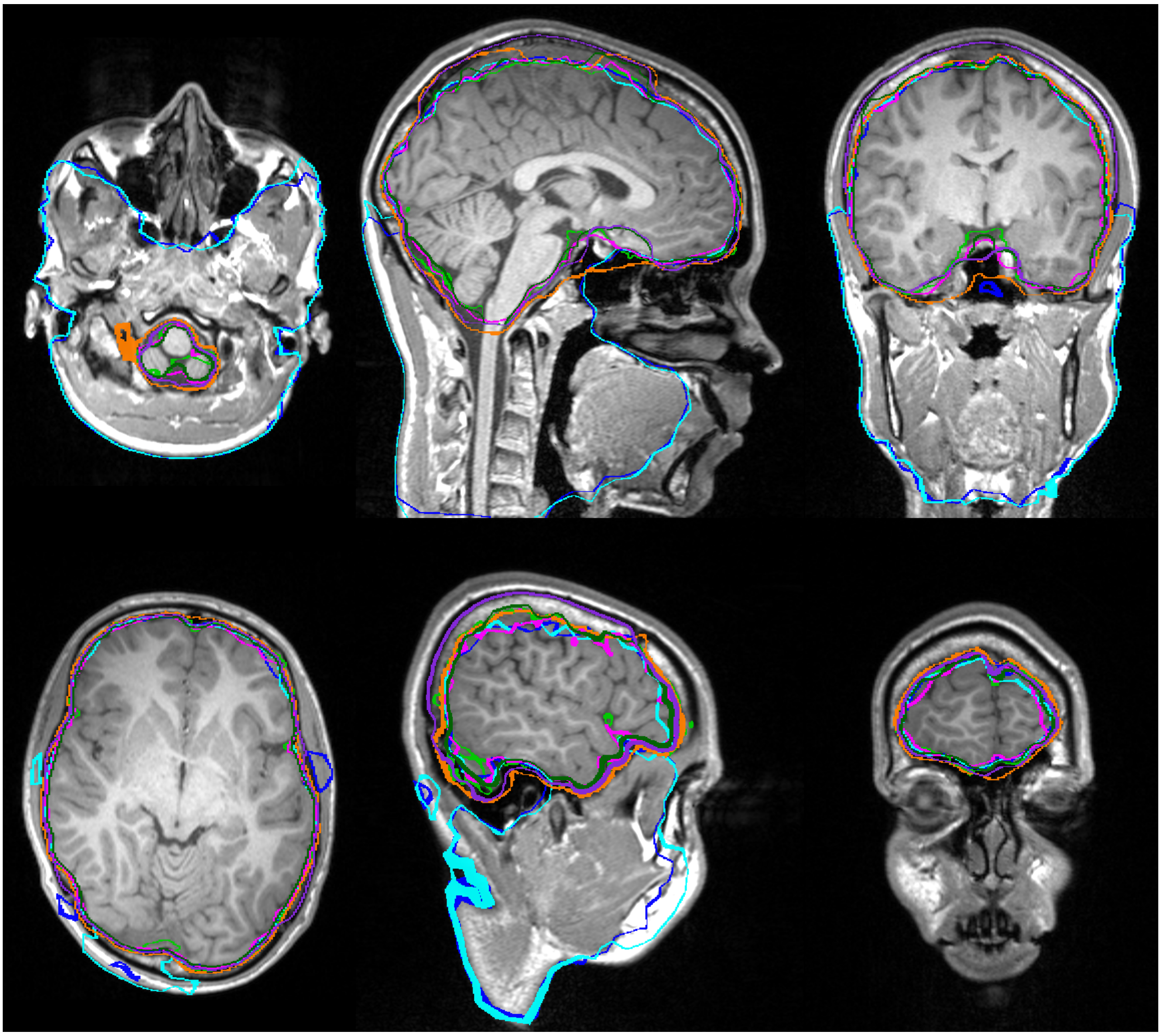

Figure 8. Adolescent cohort - typical example (median false negative score from MBWSS). VBM 8 graph cuts, MBWSS stage 1,MBWSS stage 2, FreeSurfer, BET, BET -R, BET -B.

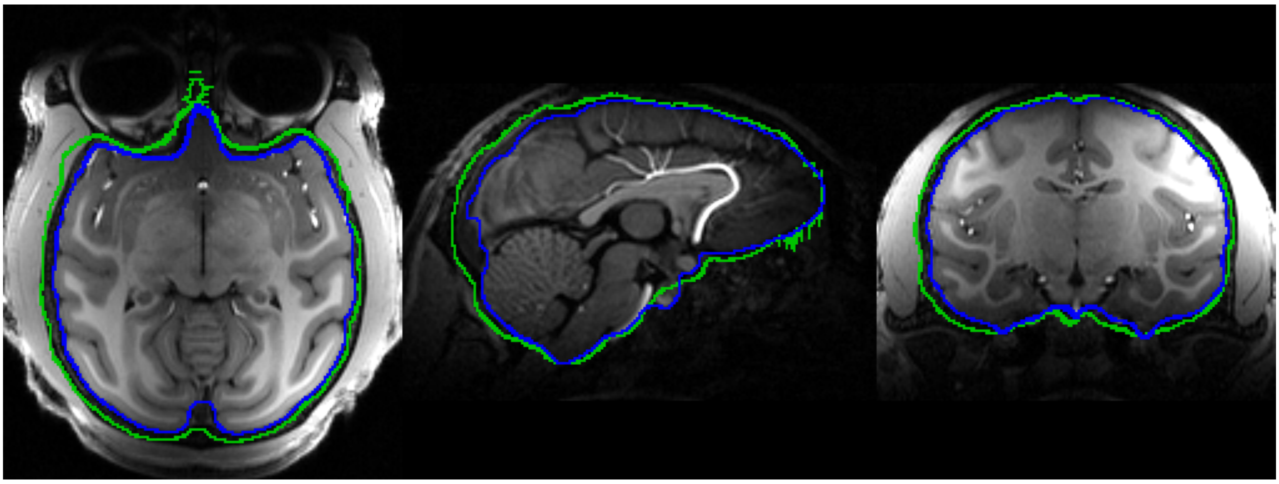

Figure 9. Macaque example 1. Manual segmentation, MBWSS stage 2.

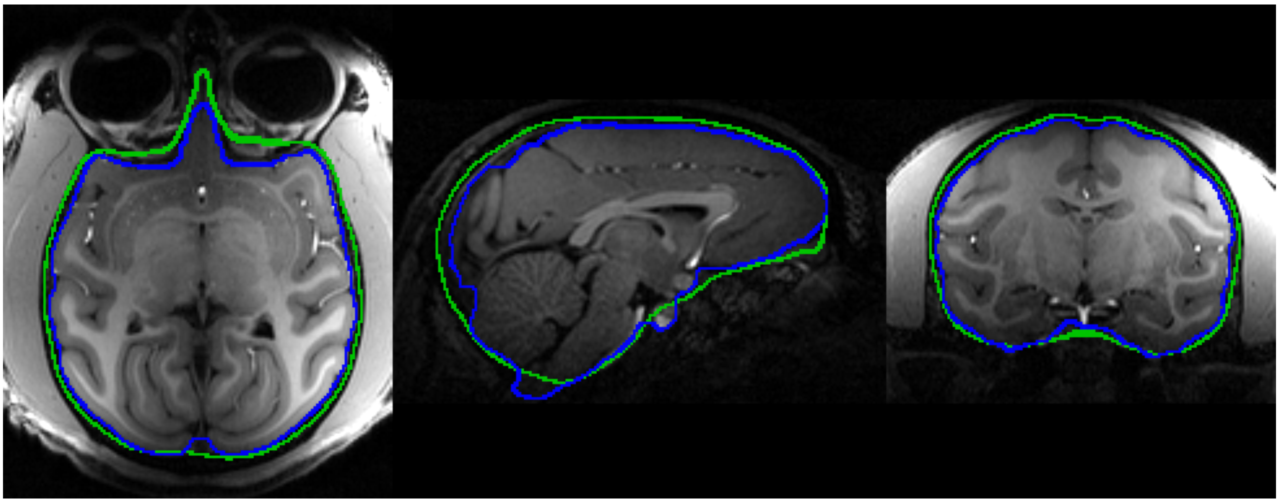

Figure 10. Macaque example 2. Manual segmentation, MBWSS stage 2.

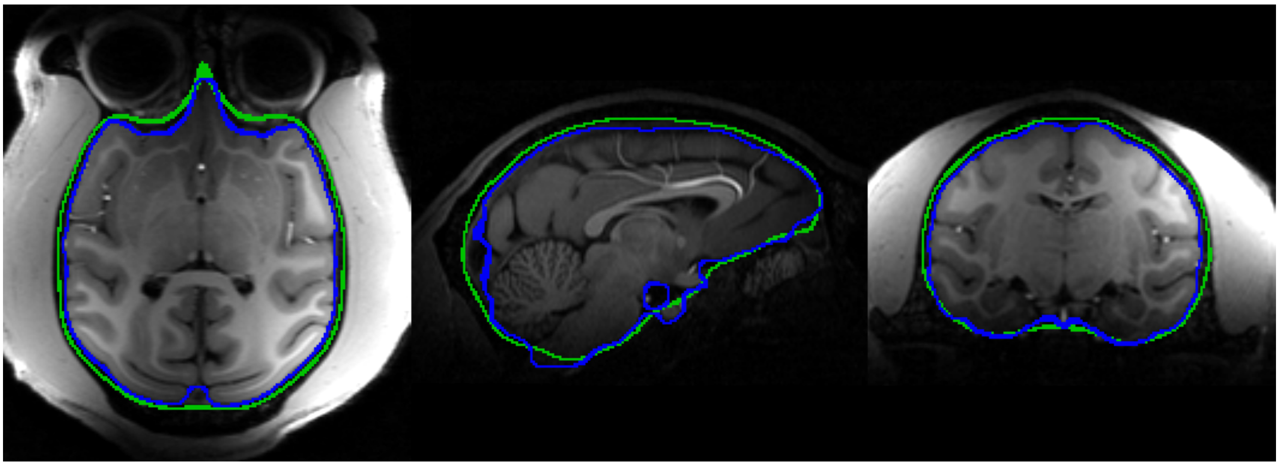

Figure 11. Macaque example 3. Manual segmentation, MBWSS stage 2.
